# Inferring pathogen dynamics from temporal count data: the emergence of *Xylella fastidiosa* in France is probably not recent

**DOI:** 10.1111/nph.15177

**Published:** 2018-04-24

**Authors:** Samuel Soubeyrand, Pauline de Jerphanion, Olivier Martin, Mathilde Saussac, Charles Manceau, Pascal Hendrikx, Christian Lannou

**Affiliations:** ^1^ BioSP INRA 84914 Avignon France; ^2^ Unit of Coordination and Support to Surveillance ANSES 69364 Lyon France; ^3^ Laboratory for Plant Health ANSES 49044 Angers France; ^4^ BIOGER INRA 78850 Thiverval‐Grignon France

**Keywords:** Bayesian inference, emerging plant pathogen, infection reservoir, introduction date, mechanistic‐statistical model, multi‐host pathogen, plant–pathogen interaction, surveillance data

## Abstract

- Unravelling the ecological structure of emerging plant pathogens persisting in multi‐host systems is challenging. In such systems, observations are often heterogeneous with respect to time, space and host species, and may lead to biases of perception. The biased perception of pathogen ecology may be exacerbated by hidden fractions of the whole host population, which may act as infection reservoirs.
- We designed a mechanistic‐statistical approach to help understand the ecology of emerging pathogens by filtering out some biases of perception. This approach, based on SIR (Susceptible–Infected–Removed) models and a Bayesian framework, disentangles epidemiological and observational processes underlying temporal counting data.
- We applied our approach to French surveillance data on *Xylella fastidiosa*, a multi‐host pathogenic bacterium recently discovered in Corsica, France. A model selection led to two diverging scenarios: one scenario without a hidden compartment and an introduction around 2001, and the other with a hidden compartment and an introduction around 1985.
- Thus, *Xylella fastidiosa* was probably introduced into Corsica much earlier than its discovery, and its control could be arduous under the hidden compartment scenario. From a methodological perspective, our approach provides insights into the dynamics of emerging plant pathogens and, in particular, the potential existence of infection reservoirs.

## Introduction

Invasions of new territories by pathogens are facilitated by the high level of connectivity of most of the world areas (Tatem *et al*., 2006; Hulme, 2009; Olsen *et al*., 2011; Fisher *et al*., 2012), despite containment and regulation strategies at the level of countries and unions of countries. In addition, global climate change allows pathogens to settle in new environments (Anderson *et al*., 2004; Jeger *et al*., 2011), which were accessible in the past only with the combined levers of migration and adaptation. For some specific threats, that is, when the pathogen effects are clearly visible or the awareness of the society is high at all levels (governmental agencies, health systems, stakeholders in forestry and agriculture, scientific communities, citizens), invasions may be detected rapidly. However, it is also common that an emerging pathogen is detected with a potentially long delay after its settlement in a new area (Jones & Baker, 2007; Waage *et al*., 2008; Faria *et al*., 2014) and the first detection may occur too late to be able to rapidly eradicate the pathogen at a reasonable socioeconomic cost.

**Figure 1:**
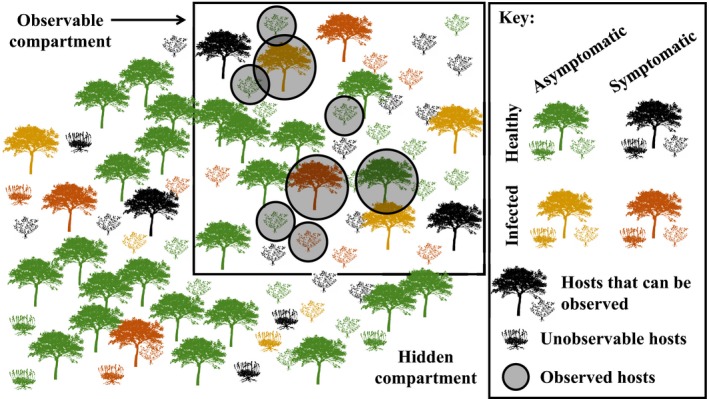
Schematic representation of the host population with an observable compartment and, complementarily, a hidden compartment (hosts outside the square). In addition, hosts are classified with respect to two other factors: healthy/infected and symptomatic/asymptomatic.

Let us consider the case in which an invading pathogen, which presents a significant threat to protected, patrimonial or cultivated plants, has been detected. Then, more or less consistent surveillance strategies can be followed to assess the sanitary situation in space and its temporal evolution, to inform decision makers, to evaluate the efficiency of eventual control measures and, more marginally but importantly, to acquire scientific knowledge. The diversity of the objectives and their time‐varying relative levels of priority lead to surveillance data that can generate biases of perception. Indeed, disease prevalence might be over‐estimated by focusing on the surveillance of areas with previously detected infected hosts. Disease incidence might be under‐estimated if a host species or a geographic region is not sampled. Disease prevalence and incidence may be under‐estimated because of the lack of power of diagnostic tests. Such biases of perception are quite common in invasion studies. For example, in a related context, the discovery rate of introduced species does not systematically reflect the actual introduction rate (Costello & Solow, 2003). In addition, for multi‐host pathogens settled in complex environments mixing cultivated, urban and wild areas, unravelling the pathogen dynamics that underlies observations may be complicated by the existence of a hidden compartment in the host population (i.e. hosts that are not observable; see Fig. 1), which may play the role of an infection reservoir (Haydon *et al*., 2002; Viana *et al*., 2014) and have an influence on the observations limited to the observable compartment.

**Figure 2:**
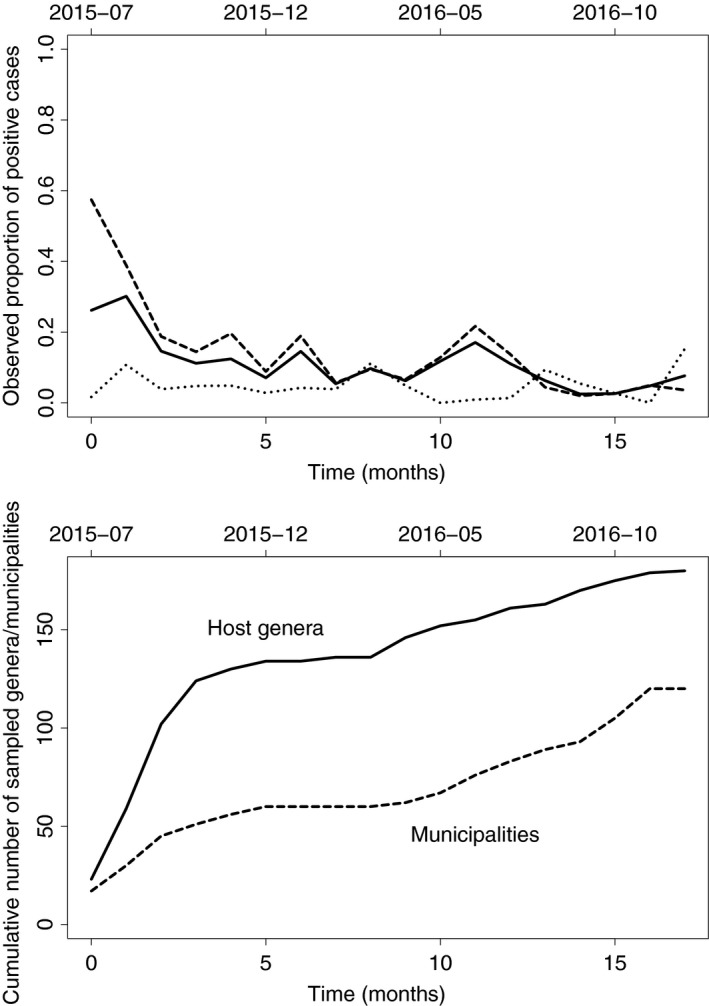
Raw data from the surveillance of *Xylella fastidiosa* in South Corsica, France, in 2015–2016. The first observation of *X. fastidiosa* in South Corsica was made in July 2015. Upper panel: observed proportion of plants positive for *X. fastidiosa* among all sampled plants (continuous line), symptomatic plants (dashed line) and asymptomatic plants (dotted line). Lower panel: cumulative counts of sampled plant genera (continuous line) and municipalities (dashed line); there are 124 municipalities in South Corsica. The list of sampled plant genera is provided in Supporting Information Notes S1 and includes a large number of wild and ornamental species.

As an illustration, let us consider the European situation of *Xylella fastidiosa*, which has been in situ detected and identified in 2013 in Italy, 2015 in France and 2016 in Spain. *Xylella fastidiosa* is a bacterium with a large range of wild and cultivated host plants, which lies in the xylem of the plant and may cause a rapid decline of its host (Purcell, 2013). *Xylella fastidiosa* is spread by insect vectors that feed on plant xylem (e.g. *Philaenus spumarius*; Saponari *et al*., 2014) and by transport of infected plants. The capacity for *X. fastidiosa* to invade new environments is facilitated by the existence of numerous strains varying in their host range and environmental preference, its multi‐host nature and the difficulty in observing the infection as a result of either a lack of symptoms or symptoms similar to those caused by other disorders (e.g. water stress). Since *X. fastidiosa* was detected in situ in South Corsica, France, during summer of 2015, a surveillance and control protocol focused on *X. fastidiosa* has been implemented and applied by local governmental agencies and other stakeholders. In this protocol, detected positive cases (as well as surrounding potential host and symptomatic plants) were destroyed to control the propagation of *X. fastidiosa*. Fig. 2 (upper panel) displays the observed proportion of positive cases, for symptomatic and asymptomatic plants, across time since the first detection. This proportion tends to decrease with time. Meanwhile, although the surveillance was initially mainly focused on host species already detected as infected in Corsica and areas surrounding positive cases, the cumulative numbers of sampled host genera and sampled municipalities were later significantly increased (see Fig. 2, lower panel) with the aim to better assess the presence of *X. fastidiosa* in terms of host range and geographic space. Thus, the decrease in the proportion of positive cases might be the consequence of (1) the destruction of hosts in foci of *X. fastidiosa* and (2) a decrease in the preference of sampling at‐risk hosts. Point (2) is a possible source of bias of perception that should be filtered out to determine which epidemic underlies the observations shown in Fig. 2. Moreover, because of the multi‐host nature of *X. fastidiosa* and the highly diverse plant population in Corsica, including large wild areas, the hidden compartment/infection reservoir hypothesis is plausible *a priori* and should be tested.

Understanding the complex ecological structures of pathogens, such as *X. fastidiosa,* is a long‐term and multidisciplinary task. Model‐based analyses of large‐scale data can contribute to such an understanding. In particular, the mechanistic‐statistical approach can help to elucidate the contributions of diverse epidemiological and observational components in data. This approach couples a mechanistic model of the temporal dynamics of the disease, a probabilistic model of the observation process and a statistical inference procedure (Soubeyrand *et al*., 2009). It allows the inference of epidemiological processes by taking into account specificities related to the observation process, including the sources of biases mentioned above.

In this article, we propose a mechanistic‐statistical framework to infer epidemics underlying temporal observations consisting of counting data collected from symptomatic and asymptomatic hosts. This framework is based on a discrete‐time Susceptible–Infected–Removed (SIR) model (Allen, 1994; Brauer *et al*., 2008) including a hidden compartment and a surveillance/control process. It allows the inference of pathogen dynamics in both the observable and hidden compartments of the host population, the estimation of the introduction date when data are collected over a post‐introduction observation window, and the prediction of the pathogen dynamics under various surveillance scenarios. The mechanistic‐statistical framework was applied to *X. fastidiosa* data collected in South Corsica. Several specifications of the model were tested and a model selection was carried out to assess whether a hidden compartment and a time‐varying preference in surveillance have to be accounted for. Results are discussed with respect to two main perspectives: the control of a multi‐host pathogen in a complex environment after its discovery and the role of infection reservoirs in sustaining epidemics.

## Materials and Methods

### Pathosystem

*Xylella fastidiosa* is a plant pathogenic bacterium dispersed by xylem‐sap‐feeding insects (Redak *et al*., 2004; Purcell, 2013; Baker *et al*., 2015), and by humans who may transport and plant infected hosts (e.g. Nunney *et al*., 2010; Nunes *et al*., 2003). *Xylella fastidiosa* is divided into several subspecies, including *X. fastidiosa* ssp. *Xylella fastidiosa* especially causing Pierce's disease in grapevine; *X. fastidiosa* ssp. *sandyi* especially causing oleander leaf scorch; *X. fastidiosa* ssp. *pauca* especially found on citrus, coffee and olive trees; and *X. fastidiosa* ssp. *multiplex* causing scorch diseases in a large range of hosts (Denancé *et al*., 2017b). Together, the different subspecies of *X. fastidiosa* cause diseases on more than 350 plant species from more than 200 genera and 70 botanical families (Gardi *et al*., 2016). The subspecies *multiplex*, which has been identified in a large majority of positive samples collected in Corsica, France (the subspecies not being identified in the other samples; Denancé *et al*., 2017b), is mostly found in temperate climates of the Americas and has been detected in Europe, not only in France but also in Spain in 2016 (European Commission, Ref. Ares(2017)3773669 – 27/07/2017; https://ec.europa.eu/food/sites/food/files/plant/docs/ph_biosec_legis_list-demarcated-union-territory_en.pdf).

*Xylella fastidiosa* has been studied especially for its pathogenicity on numerous host species, including plants with economic importance, but the interactions between *X. fastidiosa* and its host species are diverse and it does not appear to cause disease in most host species (Almeida & Nunney, 2015). Hence, asymptomatic infections not necessarily leading to disease development might be frequent, in particular in environments with high plant diversity, and might complicate the observation of *X. fastidiosa* in all its dimensions. This complication is increased by the capacity of *X. fastidiosa* to be transmitted by insect vectors (sharpshooter leafhoppers and spittlebugs), which are distributed worldwide in tropical and temperate climates and seem to be nonspecific, that is able to transmit diverse *X. fastidiosa* subspecies, but whose transmission efficiency is the outcome of complex vector–plant–pathogen–environment interactions (Almeida & Nunney, 2015). Thus, the presence of *X. fastidiosa* in an environment can translate into very diverse situations, including situations in which the bacteria can remain unseen for some (long) time.

### The Corsican environment

Corsica is an island in the north‐west of the Mediterranean Sea, characterized by warm summers and mild winters. It is covered by a large proportion of natural and semi‐natural habitats: wild heathlands and forests cover 44% and 30%, respectively, whereas agricultural areas and urban areas cover 12% and 2%, respectively (Corine Land Cover Inventory, 2012, http://land.copernicus.eu/faq/about-data-access).

Despite anthropic stress and an insular nature, Corsica has a high level of plant biodiversity and is one of the refugial areas in the Mediterranean region (Médail & Diadema, 2006; Jeanmonod *et al*., 2011). Numerous potential *X. fastidiosa* host species listed by Gardi *et al*. (2016) are present in Corsica, in the wild, urban and agricultural areas. In addition, at least 12 potential vector species have been reported in Corsica (Germain, 2016).

### Data

The French administration decided that an enhanced surveillance of *X. fastidiosa* was necessary after its detection in July 2015 from a *Polygala myrtifolia* population growing in Propriano, in the south‐west of the Island (the strategy was described in official plans DGAL/SDQSPV/2017‐653 and DGAL/SDQSPV/2017‐39; see https://info.agriculture.gouv.fr/gedei/site/bo-agri/instruction-2017-653 and https://info.agriculture.gouv.fr/gedei/site/bo-agri/instruction-2017-39). Samples from both symptomatic and asymptomatic plants were collected throughout the country and analysed in the plant health laboratory of the French Agency for Food, Environmental and Occupational Health and Safety (ANSES) and, from November 2015, in certified laboratories. Detection of *X. fastidiosa* in collected samples was performed with a real‐time PCR (Denancé *et al*., 2017b; technical reference: ANSES/LSV/MA039 version 1, October 2015; https://www.anses.fr/fr/system/files/ANSES_MA039_Xylellafastidiosa_final.pdf). Samples analysed as positives in certified laboratories were confirmed by the plant health laboratory of ANSES. Data on samples, their locations and the results of the PCR have been centralized in a database managed by the ANSES unit for coordination and support to surveillance, after a verification of data quality.

We extracted from the database those data which were collected from the French department Corse‐du‐Sud (i.e. South Corsica) between July 2015 and December 2016. We restricted the dataset to Corse‐du‐Sud because *X. fastidiosa* has been mostly found in this part of Corsica (the pathogen having a sparse distribution in Haute‐Corse, that is, the other department of Corsica, see Supporting Information Fig. S1, as well as in the south‐east of mainland France). Table S1 provides the counts, on a monthly basis, of sampled plants and infected plants by differentiating symptomatic and asymptomatic plants. These data were used to fit the competing models presented below.

### Models

We built a mechanistic‐statistical model based on an SIR architecture including a submodel of the controlled epidemic process and a submodel of the observation process. The control in the epidemic process results from the observation of positive cases, which are destroyed and therefore subtracted from the overall disease incidence. Below, we present the model outlines. Notes S2 and Table S2 provide details on the model construction.

In the model, time (denoted by *t*) is discrete and takes values in the set of integers (in the application, the time unit is 1 month). By convention, the time of the first observation is *t *=* *0, and the date of introduction is *t *= *t*_0_. Before *t*_0_, the total number of susceptible hosts is $N_{0}\in N^{*}$ and the proportion of the host population that is observable is ϕ∈[0,1] (there is no hidden compartment if ϕ=1). At *t*_0_, $I_{0}\in N^{*}$ infected hosts are introduced in both the observable and hidden compartment in proportions ϕ and 1−ϕ, respectively.

The submodel of the controlled epidemic process describes the discrete‐time dynamics followed by the counts of susceptible and infected hosts, and makes the distinction between these counts in the observable compartment (say *S*_O_(*t*) and *I*_O_(*t*)) on the one hand, and these counts in the hidden compartment (say *S*_H_(*t*) and *I*_H_(*t*)) on the other. This distinction does not imply independence: we assume that all infected hosts contribute to new infections in both compartments, irrespective of the compartments to which they belong. Thus, the disease dynamics in the two compartments are dependent, and the hidden compartment can play the role of infection reservoir. In the model, new infections are governed by a sort of discrete‐time renewal equation, parameterized by the infection strength parameter *w *>* *0. Infected hosts are affected by a mortality rate ρ∈[0,1] and are replaced by susceptible hosts if they have not been detected by the surveillance system. Infected hosts detected by the surveillance system are removed and replaced by resistant hosts immediately after their detection.

The assumptions made above are mathematically formalized as follows:

$$\left(\begin{array}{c} S_{O}\left(t\right)\\ S_{H}\left(t\right)\\ I_{O}\left(t\right)\\ I_{H}\left(t\right) \end{array}\right)=\left\{\begin{array}{c} \left(\begin{array}{c} \left\lfloor \phi N_{0}\right\rceil \\ \left\lfloor \left(1-\phi \right)N_{0}\right\rceil \\ 0\\ 0 \end{array}\right)\;\;\;\;\;\;\;\;\;\;\;\;\text{if}\;t<t_{0}\\ \left(\begin{array}{c} \left\lfloor \phi N_{0}\right\rceil -\left\lfloor \phi I_{0}\right\rceil \\ \left\lfloor \left(1-\phi \right)N_{0}\right\rceil -\left\lfloor \left(1-\phi \right)I_{0}\right\rceil \\ \left\lfloor \phi I_{0}\right\rceil \\ \left\lfloor \left(1-\phi \right)I_{0}\right\rceil \end{array}\right)\;\;\;\;\;\;\;\;\;\text{if}\;t=t_{0}\\ \left(\begin{array}{c} S_{O}\left(t-1\right)\\ S_{H}\left(t-1\right)\\ I_{O}\left(t-1\right)\\ I_{H}\left(t-1\right) \end{array}\right)+\left(\begin{array}{c} \left\lfloor \rho \left\{I_{O}\left(t-1\right)-I_{\mathrm{obs}}\left(t-1\right)\right\}\right\rceil -I_{O}^{*}\left(t\right)\\ \left\lfloor \rho I_{H}\left(t-1\right)\right\rceil -I_{H}^{}*\left(t\right)\\ -I_{\mathrm{obs}}\left(t-1\right)-\left\lfloor \rho \left\{I_{O}\left(t-1\right)-I_{\mathrm{obs}}\left(t-1\right)\right\}\right\rceil +I_{O}^{}*\left(t\right)\\ -\left\lfloor \rho I_{H}\left(t-1\right)\right\rceil +I_{H}^{}*\left(t\right) \end{array}\right)\;\text{if}\;t>t_{0} \end{array}\right.$$

where ⌊·⌉ is the rounding operator introduced to obtain integer values for (*S*_O_(*t*), *S*_H_(*t*), *I*_O_(*t*), *I*_H_(*t*)); *I*_obs_(*t*−1) is the number of (symptomatic and asymptomatic) infected hosts detected at time *t*−1; $I_{O}^{*}\left(t\right)$ and $I_{H}^{*}\left(t\right)$ are counts of new infected hosts in the observable and hidden compartments, respectively, and satisfy:

$$\begin{array}{cc} I_{O}^{*}\left(t\right) & =\left\lfloor \min\left\{1,w\frac{I(t-k)}{N(t-k)}\right\}S_{O}\left(t-1\right)\right\rceil \\ I_{H}^{*}\left(t\right) & =\left\lfloor \min\left\{1,w\frac{I(t-k)}{N(t-k)}\right\}S_{H}\left(t-1\right)\right\rceil \end{array}$$

In the application, we set *k *=* *12 months such that *w* measures the contribution of the overall disease prevalence 1 yr in the past to new infections at time *t*. Setting *k *=* *12 allows the inference of an eventual annual periodicity. More flexible forms for $I_{O}^{*}\left(t\right)$ and $I_{H}^{*}\left(t\right)$ are presented in Notes S2, but the additional model flexibility leads to convergence issues in the estimation algorithm given the information contained in the data at our disposal, and we therefore rely on the simple forms presented above.

By definition, the observation process only applies to the observable compartment. Thus, the model for the numbers $I_{\mathrm{obs}}^{†}\left(t\right)$ and $I_{\mathrm{obs}}^{\emptyset }\left(t\right)$ of symptomatic and asymptomatic observed infected hosts ($I_{\mathrm{obs}}^{†}+I_{\mathrm{obs}}^{\emptyset }=I_{\mathrm{obs}}$) takes as input variables *S*_O_(*t*), *I*_O_(*t*) and the numbers of sampled symptomatic and asymptomatic hosts, but not *S*_H_(*t*) and *I*_H_(*t*). In our approach, $I_{\mathrm{obs}}^{†}\left(t\right)$ and $I_{\mathrm{obs}}^{\emptyset }\left(t\right)$ are drawn in hypergeometric distributions taking into account the rate ϵ∈[0,1] of false negatives in the diagnostic test, and a time‐varying preference in sampling at‐risk hosts introduced in the model with the function t↦g(t). The sub‐model of the observation process also includes parameters $\gamma_{1}$ and $\gamma_{2}$ lying in [0, 1], which are the proportions of symptomatic hosts among infected and susceptible hosts, respectively, belonging to the observable compartment. Thus, when the counts of symptomatic and asymptomatic observed hosts at time *t*, say $N_{\mathrm{obs}}^{†}\left(t\right)$ and $N_{\mathrm{obs}}^{\emptyset }\left(t\right)$, are positive:

$$\begin{array}{cc} I_{\mathrm{obs}}^{†}\left(t\right) & ∼\text{Hypergeometric}\\ & \left(\right.\left\lfloor \left(1-\epsilon \right)I_{O}^{†}\left(t\right)\right\rceil ,S_{O}^{†}\left(t\right)+\left\lfloor \epsilon I_{O}^{†}\left(t\right)\right\rceil ,N_{\mathrm{obs}}^{†}\left(t\right))\\ I_{\mathrm{obs}}^{\emptyset }\left(t\right) & ∼\text{Hypergeometric}\\ & \left(\right.\left\lfloor \left(1-\epsilon \right)I_{O}^{\emptyset }\left(t\right)\right\rceil ,S_{O}^{\emptyset }\left(t\right)+\left\lfloor \epsilon I_{O}^{\emptyset }\left(t\right)\right\rceil ,N_{\mathrm{obs}}^{\emptyset }\left(t\right)) \end{array}$$

where the hypergeometric distribution is parameterized by the numbers of successes and defaults in the population and the number of draws; $I_{O}^{†}\left(t\right)$ and $S_{O}^{†}\left(t\right)$ are the numbers of symptomatic hosts at time *t* in the observable compartment that are infected and susceptible, respectively; and $I_{O}^{\emptyset }\left(t\right)$ and $S_{O}^{\emptyset }\left(t\right)$ are the numbers of asymptomatic hosts at time *t* in the observable compartment that are infected and susceptible, respectively, and that are considered as at‐risk. These numbers satisfy:

$$\begin{array}{cc} & I_{O}^{†}\left(t\right)=\gamma_{1}I_{O}\left(t\right)\\ & I_{O}^{\emptyset }\left(t\right)=I_{O}\left(t\right)-I_{O}^{†}\left(t\right)=\left(1-\gamma_{1}\right)I_{O}\left(t\right)\\ & S_{O}^{†}\left(t\right)=g\left(t\right)\gamma_{2}S_{O}\left(t\right)\\ & S_{O}^{\emptyset }\left(t\right)=g\left(t\right)\left(1-\gamma_{2}\right)S_{O}\left(t\right) \end{array}$$

where *g*(*t*) is the time‐varying proportion of susceptible hosts (both symptomatic and asymptomatic) in the observable compartment that are considered as at‐risk, that is, that are likely to be sampled (note that all infected hosts in the observable compartment are considered as at‐risk and are consequently likely to be sampled). It should be noted that the fraction ϵ of infected hosts is removed from the number of successes in each hypergeometric distribution and added to the number of defaults to take into account the risk of false negatives.

In the hypergeometric distributions, a given number of hosts are sampled in a finite population of infected and susceptible hosts, up to the false‐negative rate, and the sampling is assumed to be uniformly random among the infected and susceptible hosts. However, the sampling may be orientated towards at‐risk hosts, and this orientation may change with time. In particular, susceptible hosts might have a reduced propensity to be sampled because of the current knowledge about the epidemic and noticeable host factors (e.g. altitude, distance to infected areas and species). We did not explicitly take into account these factors, but we handled their effects by introducing into the model the function *g* that takes values of [0, 1] and reduces the number of susceptible hosts appearing in each hypergeometric distribution. More precisely, the function *g* gives the time‐varying proportion of the susceptible hosts in the observable compartment which can be sampled. These hosts, together with infected hosts in the observable compartment, are called *at‐risk* hosts. The function *g* is parameterized by β_1_ and β_2_ in [0, 1], which gives, respectively, the values of *g* at the first and last times of observation.

In the Results section, we use the preference in sampling at‐risk hosts, which is defined as the ratio Pref(*t*)=1/(1 + *g*(*t*)) and gives the probability of sampling the infected host within a set of two hosts, one being infected and the other being healthy.

In the application, we consider eight competing models, denoted $M_{1},\ldots ,M_{8}$, which are different instances of the modelling framework described above. They correspond to different specifications concerning the existence of a hidden compartment and the preference in sampling at‐risk hosts. Table 1 provides the model specificities.

**Table 1:** Specifications of the hidden compartment and the preference in sampling for models $M_{1},\ldots M_{8}$; it should be noted that models $M_{6},M_{7}\;\text{and}\;M_{8}$ have different prior distributions for the parameter ϕ

Preference in sampling	Hidden compartment	
	None ϕ=1	Fraction of the whole population ϕ∈[0,1]
None g≡1	$M_{1}$	$M_{4}$
At‐risk, constant g≡cst∈[0,1]	$M_{2}$	$M_{5}$
At‐risk, linearly varying	$M_{3}$	$M_{6}$ (uniform prior in [0, 1] for ϕ)
*g*: linear function		$M_{7}$ (*a priori* large value for ϕ)
with values in [0, 1]		$M_{8}$ (*a priori* small value for ϕ)

### Bayesian estimation and model selection

Models $M_{1},\ldots ,M_{8}$ are parameterized by:

$$\theta =(t_{0},N_{0},I_{0},\rho ,w,\phi ,\gamma_{1},\gamma_{2},\beta_{1},\beta_{2},\epsilon )$$

In models $M_{1},M_{2}\;\text{and}\;M_{3}$, the proportion of the host population that is observable is fixed at ϕ=1. In models $M_{1}$ and $M_{4}$, $g\equiv \beta_{1}=\beta_{2}=1$. In models $M_{2}$ and $M_{5}$, $g\equiv \beta_{1}=\beta_{2}$, where $\beta_{1}$ has to be estimated.

More or less informative priors were chosen depending on the available knowledge about the parameters. Prior distributions are specified and motivated in Notes S2 and Table S3, and are briefly described in what follows. The prior for the introduction date *t*_0_ was relatively vague (uniform prior over the 50 yr preceding the first detection of *X. fastidiosa* in Corsica). The total number of susceptible host units *N*_0_ at *t*_0_ had a prior mean of 5.5 million and a range between 1.9 and 13.3 million (prior quantiles of order 0.025 and 0.975). The number *I*_0_ of introduced infected hosts at *t*_0_ was set at a fixed value in all models because of some identifiability issues. This is the only parameter that we did not infer. We set *I*_0_ = 10, which amounts to the assumption that the epidemic began with the introduction of a small batch of infected plants and that subsequent introductions did not significantly impact the overall curse of the epidemic. Notes S3 and Figs S2 and S3 provide an analysis of the impact of the value of *I*_0_ on the inference output. The prior distribution for the mortality rate ρ was chosen to encompass significantly different mortality dynamics (roughly, from 50% of death in the first year of infection to 50% of death in the first 7.7 yr of infection). A vague uniform prior over [0, 10] was used for *w*. For the proportions $\phi ,\gamma_{1},\gamma_{2},\beta_{1}\;\text{and}\;\beta_{2}$, we chose vague uniform priors over [0, 1], except in the following cases: for models $M_{1}$, $M_{2}$ and $M_{3}$ without hidden compartment, ϕ was equal to 1; for models $M_{7}$ (with an *a priori* small hidden compartment) and $M_{8}$ (with an *a priori* large hidden compartment), the prior for ϕ was a beta distribution with parameter vectors equal to (4, 1) and (1, 4), respectively; for models $M_{1}$ and $M_{4}$, $\beta_{1}=\beta_{2}=1$; for models $M_{2}$ and $M_{5}$, $\beta_{1}$ was *a priori* uniform over [0, 1] and $\beta_{2}=\beta_{1}$. Finally, the false‐negative rate ϵ was *a priori* uniformly distributed over [0,0.2], that is, ϵ was *a priori* rather low, but could take non‐negligible values.

Parameters were estimated with an Markov chain Monte Carlo (MCMC) algorithm with Metropolis–Hastings updates. Three chains were run for each model to check the convergence of the algorithm, and were merged to obtain large posterior samples of parameters. Parameters were updated by blocks with a Gaussian proposal distribution centred around the current parameter values (the variances in the proposal distribution were tuned to obtain rapid algorithm convergence). For each MCMC run, we performed $2\times 10^{7}$ iterations, applied a burnin of $4\times 10^{6}$ iterations, and subsampled the rest of the chain every 2000 iterations. Thus, posterior samples were formed by 24 000 vectors of parameter values.

Model selection was performed with respect to several criteria: the Akaike's information criterion (AIC), the Bayesian information criterion (BIC), the deviance information criterion (DIC) proposed by Spiegelhalter *et al*. (2002), the DIC modification proposed by Gelman *et al*. (2014, Chapter 7), the DIC modification proposed by Ando (2011) and the Bayes factor computed from the harmonic mean of the likelihood values (Kass & Raftery, 1995).

## Results

### Dualism in model selection

Among the competing models $M_{1}-M_{6}$, the best models are those with a preference in sampling at‐risk hosts, which varies across time (Table 2). In addition, the incorporation in the model of a hidden compartment seems to be useless based on the diverse selection criteria. It should be note that not selecting a model with a hidden compartment does not mean that the hidden compartment does not exist, but tends to indicate that the hidden compartment, if any, has a negligible influence on the observations (see the Discussion section).

**Table 2:** Selection criteria computed for models with different specifications for the hidden compartment and the preference in sampling

Hidden compartment	Preference in sampling	Model	Log_L_	AIC	BIC	DIC‐S	DIC‐G	DIC‐A	Bayes factor
None	None	$M_{1}$	−224	463	512	436	477	411	<10^−4^
	At‐risk, constant	$M_{2}$	−229	476	539	417	475	368	<10^−4^
	At‐risk, varying	$M_{3}$	−197	**412**	**475**	**356**	**412**	**308**	1.00
Fraction of the whole population	None	$M_{4}$	−224	465	520	461	481	460	<10^−4^
	At‐risk, constant	$M_{5}$	−229	478	547	388	477	309	<10^−4^
	At‐risk, varying	$M_{6}$	−197	415	484	NA	416	NA	0.80
*A priori* small fraction	At‐risk, varying	$M_{7}$	−197	414	484	363	414	321	0.08
*A priori* large fraction	At‐risk, varying	$M_{8}$	−199	418	488	399	415	392	**1.41**

Log_L_ is the log‐likelihood, AIC is the Akaike’s information, BIC is the Bayesian information criterion, DIC‐S, DIC‐G and DIC‐A are the deviance information criteria of Spiegelhalter *et al*. (2002), Gelman *et al*. (2014) and Ando (2011). DIC‐S and DIC‐A cannot be calculated for model $M_{6}$, for which the posterior mean of the parameter vector is unlikely because of the multimodality of the posterior (this is indicated in the table by NA, which stands for not available). $M_{8}$ is selected as the best model by the Bayes factor, whereas $M_{3}$ is selected by the other criteria (figures in bold).

**Figure 3:**
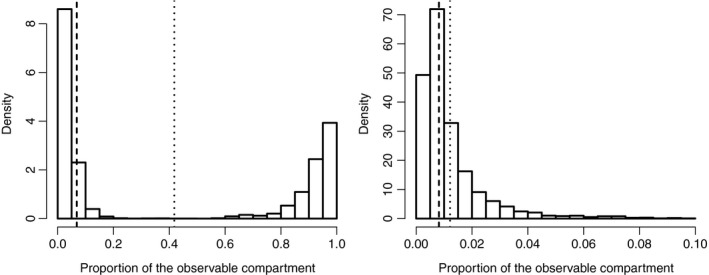
Posterior distribution of the proportion ϕ of the observable compartment under models $M_{6}$ (left) and $M_{8}$ (right). The dotted and dashed lines indicate the posterior mean and median of ϕ, respectively.

A closer look at the hidden compartment hypothesis leads to an unexpected result: under model $M_{6}$ (which contains a hidden compartment, a vague prior for ϕ and a varying preference in sampling at‐risk hosts), the proportion ϕ of the observable compartment has a clearly bimodal posterior distribution (Fig. 3, left), with large probabilities for values close to either zero (i.e. most of the hosts are hidden) or one (i.e. most of the hosts are observable); the latter case is well approximated by model $M_{3}$, in which ϕ=1. We investigated this characteristic by generating two additional competing models differing from model $M_{6}$ with respect to the prior distribution of ϕ : we changed the uniform prior into a beta prior with shape parameters (4, 1) for model $M_{7}$ and (1, 4) for model $M_{8}$. Thus, under $M_{7}$ ($M_{8}$), the prior mean of ϕ is 0.8 (0.2) and the hidden compartment is *a priori* a small (large) fraction of the whole host population. Based on the Bayes factor, model $M_{8}$ with a large hidden compartment is the best model and, a posteriori, the hidden compartment represents *c*. 99% of the whole host population (Fig. 3, right; 95%‐posterior interval: [95%; 100%]).

**Figure 4:**
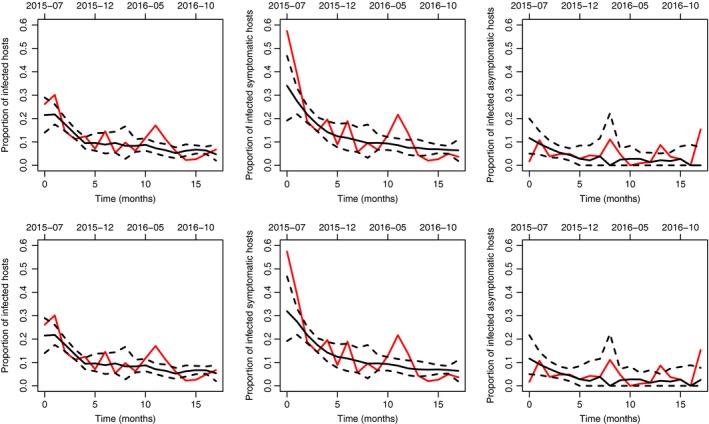
Proportion of infected hosts across time under models $M_{3}$ (upper) and $M_{8}$ (lower). The proportion is computed for all hosts (left), symptomatic hosts (centre) and asymptomatic hosts (right). Red curve, observed proportion; black continuous curve, posterior median; black dashed curves, pointwise posterior quantiles of order 0.025 and 0.975.

This dualism in the model selection led us to present in what follows the inferences obtained under both models $M_{3}$ (without a hidden compartment) and $M_{8}$ (with a hidden compartment), which similarly fit the raw data obtained from the surveillance of *X. fastidiosa* in South Corsica (see Fig. 4).

### Two scenarios in the past

**Figure 5:**
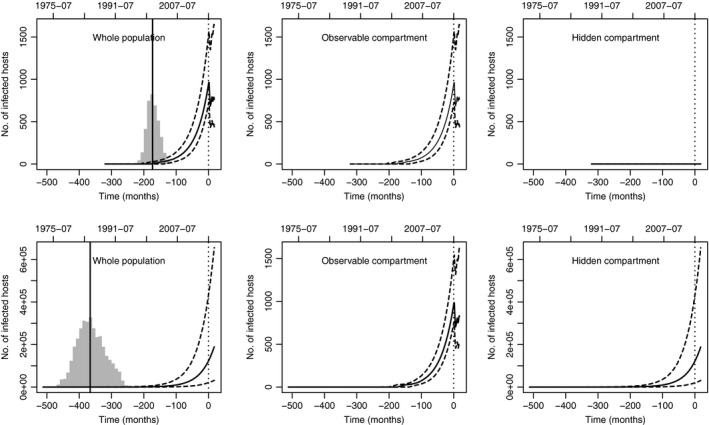
Posterior medians, 0.025 quantiles and 0.975 quantiles of the past numbers of infected hosts in the whole host population (left), the observable compartment (centre) and the hidden compartment (right) under models $M_{3}$ (upper) and $M_{8}$ (lower); the median is given by the continuous curve, the quantiles by the dashed curves. The number of infected hosts in the hidden compartment is zero under model $M_{3}$ as this compartment is empty. In the left panels, the grey histograms and the continuous vertical line give the posterior distributions of the introduction date and its posterior median under each model. The dotted vertical line gives the date of the first observation.

The inferences obtained under models $M_{3}$ and $M_{8}$ correspond to two different scenarios mostly diverging in terms of the introduction date (*t*_0_) and total number of infected hosts. In scenario 1 (model $M_{3}$), the introduction occurred around 2001, and the infected host units ranged from 400 to 1700 at the end of 2016. In scenario 2 (model $M_{8}$), the introduction occurred around 1985, and the infected host units ranges from 30 000 to 660 000 at the end of 2016 (see Fig. 5; Tables S4, S5).

Interestingly, the posterior of the number *N*_0_ of susceptible host units at the introduction date is approximately the same under models $M_{3}$ and $M_{8}$. Thus, the two scenarios are based on a similar description of the host population except for the fact that a large fraction of the population is hidden in scenario 2. Consequently, the difference in the number of infected hosts provided above translates into a difference in proportions: a very small proportion (≈ 3‱) of the host population is infected in scenario 1, much smaller than the corresponding proportion (≈5%) in scenario 2 (Table S5; Fig. S4).

**Figure 6:**
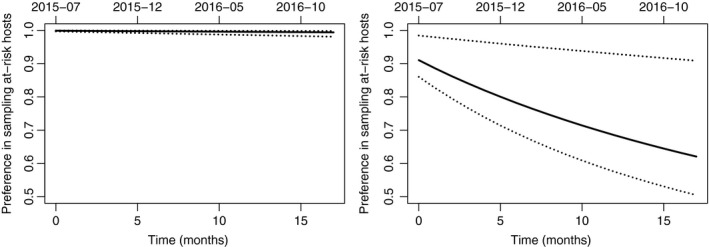
Posterior medians (continuous curve), 0.025 quantiles and 0.975 quantiles (dotted curves) of the preference in sampling at‐risk hosts across time under model $M_{3}$ (left) and $M_{8}$ (right).

The size *N*_0_ is not the only parameter similarly estimated with models $M_{3}$ and $M_{8}$. Indeed, we obtained consistent estimations of the mortality rate (ρ), the infection strength (*w*), the proportions of symptomatic hosts in the observable compartment ($\gamma_{1}$ and $\gamma_{2}$) and the false‐negative rate (ϵ) (see Figs S5, S6). Hence, the two scenarios share several epidemiological and observational features. There is however an observational feature that varies: the preference in sampling at‐risk hosts. This preference decreases in both scenarios, but the magnitude of decrease is different. In scenario 1, where the observable compartment is huge as it coincides with the whole population, Pref(*t*) remains very high (it decreases from nearly 0.999 to 0.995). In scenario 2, Pref(*t*), which applies only to the observable compartment, decreases from nearly 0.9 to 0.6 (see Fig. 6). We will see below that this preference in sampling at‐risk hosts may be a crucial lever for controlling the disease dynamics.

### Implications for the future

We have previously highlighted differences and similarities in the two past scenarios for the *X. fastidiosa* dynamics in South Corsica. When one looks at the future, the models $M_{3}$ and $M_{8}$ provide significantly different outputs. As demonstrated below, the hidden compartment in model $M_{8}$ plays the role of infection reservoir, which would make the control of the disease difficult.

**Figure 7:**
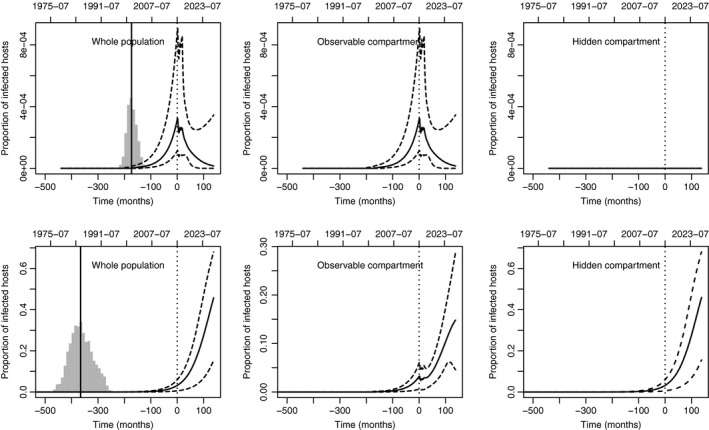
Posterior medians, 0.025 quantiles and 0.975 quantiles of the past and future proportions of infected hosts in the whole host population (left), the observable compartment (center) and the hidden compartment (right) under models $M_{3}$ (upper) and $M_{8}$ (lower). In the prediction part of the curves, 800 symptomatic plants and 200 asymptomatic plants were sampled per month and Pref(*t*) = 0.995 with model $M_{3}$ and Pref(*t*) = 0.6 with model $M_{8}$ (see Fig. 5 for additional details on plot construction).

Fig. 7 shows, for the next 10 yr, the predictions of the proportion of infected hosts in the whole population, the observable compartment and the hidden compartment under models $M_{3}$ (top panels) and $M_{8}$ (bottom panels). These predictions were made with a constant (but reinforced) surveillance effort and a constant preference in sampling at‐risk hosts: 800 symptomatic plants and 200 asymptomatic plants were sampled per month (these values are among the highest values encountered in the past surveillance; see Table S1), and Pref(*t*) = 0.995 with model $M_{3}$ and Pref(*t*) = 0.6 with model $M_{8}$ (these values were those estimated at the end of the sampling period; see Fig. 6).

With such a characterization of the surveillance, *X. fastidiosa* could be brought to low levels under model $M_{3}$ (the oscillating curse of the epidemic during the actual surveillance period, from month 0 to 17, vanishes thanks to the reinforced surveillance), but should continue to increase under model $M_{8}$, even in the observable compartment. This noticeable difference occurs although we estimated approximately the same number of infected hosts in the observable compartment with both models (Fig. 5). Thus, under model $M_{8}$, we can see the positive effect of the hidden compartment on the development of *X. fastidiosa* and, consequently, its role as infection reservoir. The effect of the hidden compartment is initially weak (we observe in the bottom centre panel of Fig. 7 a nearly constant prevalence in the observable compartment from month 0 to month 40, the hidden compartment and the reinforced surveillance generating opposite but comparable forces). After month 40, the continuous growth of the prevalence in the hidden compartment, which is not controlled, has a larger impact on the infection dynamics than does the reinforced surveillance and, consequently, the prevalence in the observable compartment significantly increases. Figs S7 and S8 provide 10‐yr predictions for diverse characterizations of the surveillance. They especially show that increasing the preference in sampling at‐risk hosts (as defined in our work) is a lever to be considered for reducing disease prevalence (and not only a source of bias of perception). Indeed, a large preference in sampling at‐risk hosts, Pref(*t*) = 1/(1 + *g*(*t*)), amounts focusing the surveillance on actually infected hosts, which are destroyed after their detection, and therefore to more efficiently reducing the disease prevalence. However, the correct way to increase Pref(*t*) is not obvious in practice: it can be increased by preferentially sampling species and areas that are known to be infected, but one must avoid simultaneously enlarging the hidden compartment. For instance, if one samples only the most infected species, then all the other infected species enter into the hidden compartment.

## Discussion

Based on temporal observations and an adapted original model, our analyses tend to show that the emergence of *X. fastidiosa* in Corsica, France, is probably not a recent story. The model selection led to two scenarios: the first with an introduction around 2001 (1998–2005) and without a hidden compartment, and the second with an introduction around 1985 (1978–1993) and a hidden compartment. The two scenarios also diverge in terms of prediction, the scenario with a hidden compartment leading to significantly more severe future epidemics irrespective of the applied control measures.

To determine which scenario is more realistic requires further data collection and analyses. In particular, evaluation of what could be the hidden compartment (e.g. wild and semi‐natural landscape components, or host species for which diagnostic tests are not done or not efficient) and sampling in this compartment are crucial to test the veracity of our second scenario. Although new data should be produced to investigate specific epidemiological questions and to better unravel the ecological structure of *X. fastidiosa* in Corsica, existing data still contain unexploited information. Indeed, our approach is only based on time series providing the symptomatic nature of sampled plants and their observed health status with respect to *X. fastidiosa*. Further analyses should be carried out to more finely exploit the spatiotemporal surveillance dataset available (e.g. spatial coordinates and species information of sampled hosts, and genetic information on bacterial strains). Such analyses should lead to more accurate results on the date of introduction and other epidemiological parameters, such as the mortality rate and the infection strength of infectious hosts. They should also provide information on processes not accounted for in our work, for instance, the dynamics of vectors (as in Bosso *et al*., 2016; White *et al*., 2017), the evolution of bacterial strains and the spatial spread of the disease. In particular, including in the analyses genetic and demographic data from North Corsica and south‐east of mainland France, where *X. fastidiosa* has been more sporadically detected, could provide crucial information on eventual multiple introductions and human‐mediated long‐distance dispersal (as in Mollentze *et al*., 2014, in the case of rabies).

Inferences made about *X. fastidiosa* are obviously constrained by the features of our model. In particular, this model explicitly incorporates a hidden compartment, but ignores spatial and species information. The explicit incorporation of the counts of susceptible and infected hosts in the hidden compartment is a way to objectively account for the time‐varying risk of infection caused by infected hidden hosts. This approach is adopted in many temporal SIR‐like models that make the distinction between different types of hosts, for example target hosts and alternate hosts, including vectors (Dobson, 2004; Allen *et al*., 2012). Such multi‐host epidemic models are often based on a system of ordinary differential equations, but can also be based on Markov processes (McCormack & Allen, 2006; Allen, 2017), as in our case.

A classical alternative modelling approach is to decompose the risk of infection into two components, the first that is dependent on the number of infected hosts in the compartment of interest (often modelled as an auto‐regressive term) and the second that is independent from this number (Held *et al*., 2006; Unkel *et al*., 2012). The second component is a way to implicitly handle alternate/hidden hosts but also environmental to factors, it is generally time‐varying, can incorporate explanatory variables and can be estimated for example, in the framework of hidden Markov models (HMMs).

Although our model takes into account various epidemiological components (observable/hidden host compartments, symptomatic/asymptomatic status of hosts, delay of infection, preference in sampling), it nevertheless ignores spatial and species information, as mentioned above. Indeed, our model is built on a mean‐field assumption (or homogeneous mixing assumption) concerning the interaction between hosts, as are many deterministic or stochastic epidemiological models (Kleczkowski & Grenfell, 1999; Keeling & Grenfell, 2000; Aparicio & Pascual, 2007; Britton *et al*., 2015): the effect of the other hosts on any host is approximated by a single average effect, irrespective of their locations and species. Obviously, this assumption is not perfectly realistic for a pathogen that can be spread by insects (mostly at short distances and certainly with heterogeneous cross‐species transmissions) and by humans (both at short and long distances and with between‐host‐species heterogeneities). Hence, it would be worthwhile assessing the inference accuracy achieved with our model for data simulated under a spatially and species‐explicit model, as predictions under mean‐field models are compared with predictions obtained by their individual‐based counterparts.

Dating pathogen emergences is a complex issue, but the integration of different sources of information can help to reduce the uncertainty. Dates of introduction of pathogens have been inferred from various types of data – for example demographic data (this article; Heiler *et al*., 2013; Soubeyrand & Roques, 2014), genomic data (Dudas & Rambaut, 2014; Nunes *et al*., 2014), archaeological data, archives and historical records (Le Floc'h, 1991; Preston *et al*., 2004; Potter *et al*., 2011) – and various analyses techniques – for example epidemiological investigations, forward simulations of population dynamic models, statistical estimation techniques, phylogenetic and phylogeographic analyses. Despite these data and techniques, origins of outbreaks generally remain uncertain (Woolhouse & Gaunt, 2007; with the exception of situations in which epidemiological investigations allowed the identification of the primary case(s)). This statement typically holds for plant pathogens arriving in regions in which the awareness is not focused on these pathogens at their introduction times. The combination of different analyses performed with different data should help to reduce the uncertainty about the origin. Concerning our case study, namely the emergence of *X. fastidiosa* in Corsica, a complementary approach based on molecular dating of a phylogenetic tree exploiting genome data provided the following mean dates of divergence between couples of French isolates and their American relatives: *c*. 1980 for strain ST6 and 1965 for strain ST7 (Denancé *et al*., 2017a). These dates can be considered as proxies or lower bounds of the introduction dates. They are relatively consistent with our second scenario (1985 (1978–1993)), and a joint analysis of demographic and genomic data could help in to refine our conclusions.

Identifying and characterizing reservoirs of infection, if any, is crucial for understanding of infectious disease dynamics, design of surveillance and control strategies, and the anticipation and prevention of future emergences (Haydon *et al*., 2002; Karesh *et al*., 2012; Bartoli *et al*., 2015). For humans, numerous pathogens have long been recognized to have environmental or animal reservoirs (the corresponding diseases being called sapronoses and zoonoses, respectively; Woolhouse & Gaunt, 2007). For agricultural plants, early examples of identification and control of infection reservoirs do exist (see, for example, the eradication of barberry, an alternate host of the wheat stem rust; Stakman, 1919), but Morris *et al*. (2007) pointed out a decade ago that pathogenic bacteria had been almost exclusively studied in agricultural contexts, neglecting environmental niches, and Burdon & Thrall (2008) designated the study of the agro‐ecological interface and its evolutionary implications as a major issue for future research. With time, plant pathogen reservoirs of various kinds have been studied (e.g. wild or weedy host plants, volunteer plants, alternate hosts, leaf litter, freshwater and snowpack; Holt *et al*., 2003; Li *et al*., 2014; Gérard *et al*., 2006; Beckstead *et al*., 2010; Fabre *et al*., 2012; Monteil *et al*., 2013; Soubeyrand *et al*., 2017), and reservoirs are today considered as important drivers in plant epidemiology. The approach developed in this article can be viewed as a data‐driven way of testing the existence (or the influence) of a reservoir during an outbreak, when data are collected only from the target population. Obviously, the influence of the reservoir has to be non‐negligible to be detected with our method, which simply exploits demographic counting data. For *X. fastidiosa* in South Corsica, we were not able to firmly determine whether or not there is a hidden compartment (viewed as an infection reservoir in our study), but our results show that this hypothesis is plausible and should be investigated in further studies. Since July 2015, more than 20 new host species have been found in Corsica (Gardi *et al*., 2016; see also https://ec.europa.eu/food/plant/plant_health_biosecurity/legislation/emergency_measures/xylella-fastidiosa/susceptible_en for updated information). This progressive discovery of host species supports the hidden compartment hypothesis. Moreover, an analysis of the demography and disease prevalence for a host such as *Cistus monspeliensis* suggests that it could be, among others, an important component of the hidden compartment. Indeed, *C. monspeliensis* is very abundant in Corsica (http://www.tela-botanica.org), in particular in wild areas; its observed infection rate is quite high (*c*. 11%), and insect vectors tend to be frequent around this host (recent molecular analyses have shown that *X. fastidiosa* is present in *c*. 20% of insect vectors *Philaenus spumarius* collected from several *C. monspeliensis* populations across Corsica; Cruaud *et al*., 2018). However, this host species has been weakly surveyed (3% of samples) in comparison with much less abundant host species, such as *Polygala myrtifolia* (12% of samples), which is an ornamental plant with an observed infection rate of 26%. Thus, the *C. monspeliensis* population is under‐represented in surveillance data and a fraction of this population, in particular in wild areas, could contribute to the hidden compartment. The evaluation of the spatial distribution of this host and its comparison with it to the spatial pattern of sampled *C. monspeliensis* in the surveillance of *X. fastidiosa* would be a first step towards the identification of a potential reservoir.

## Author contributions

S.S. conceived the ideas and designed the methodology; S.S., P.d.J., O.M. and M.S. prepared and analysed data; S.S., P.d.J., O.M., M.S., C.M., P.H. and C.L. discussed the objectives of the study at an early stage and commented on the results; S.S. led the writing of the manuscript. All authors contributed critically to the drafts and gave final approval for publication.

## Supporting information

Please note: Wiley Blackwell are not responsible for the content or functionality of any Supporting Information supplied by the authors. Any queries (other than missing material) should be directed to the *New Phytologist* Central Office.

**Fig. S1** Locations of *Xylella fastidiosa* positive and negative samples in Corsica, France, between July 2015 and December 2016.
**Fig. S2** Prior and posterior distributions of *I*_0_ under model $M_{8}$ when *I*_0_ is not fixed.
**Fig. S3** Posterior means and quantiles of parameters of model M_8_ obtained for various values of *I*_0_.
**Fig. S4** Posterior medians, 0.025 quantiles and 0.975 quantiles of the past proportions of infected hosts under models $M_{3}$ and $M_{8}$.
**Fig. S5** Marginal posterior distributions of the parameters of model $M_{3}$.
**Fig. S6** Marginal posterior distributions of the parameters of model $M_{8}$.
**Fig. S7** Posterior medians, 0.025 quantiles and 0.975 quantiles of the future proportions of infected hosts under model $M_{3}$ and different surveillance scenarios.
**Fig. S8** Posterior medians, 0.025 quantiles and 0.975 quantiles of the future proportions of infected hosts under model $M_{8}$ and different surveillance scenarios.
**Table S1** Monthly surveillance data in South Corsica
**Table S2** Specifications of the hidden compartment and the preference in sampling for models $M_{1},\ldots ,M_{8}$, with mathematical expressions of the function *g*
**Table S3** Prior distributions of parameters
**Table S4** Posterior means, medians, 0.025 quantiles and 0.975 quantiles of parameters of models $M_{3}$ and $M_{8}$
**Table S5** Posterior means, medians, 0.025 quantiles and 0.975 quantiles of the introduction year and the number/proportion of infected hosts in December 2016 under models $M_{3}$ and $M_{8}$
**Notes S1** List of plant genera sampled in South Corsica from July 2015 to December 2016.
**Notes S2** Detailed model description.
**Notes S3** Impact of the choice of the number *I*_0_ of introduced infected hosts on the estimation of the other model parameters.

## References

[nph15177-bib-0003] Allen L , Brown V , Jonsson C , Klein S , Laverty S , Magwedere K , Owen J , Van Den Driessche P . 2012 Mathematical modeling of viral zoonoses in wildlife. Natural Resource Modeling 25: 5–51. 22639490 10.1111/j.1939-7445.2011.00104.x PMC3358807

[nph15177-bib-0001] Allen LJ . 1994 Some discrete‐time SI, SIR, and SIS epidemic models. Mathematical Biosciences 124: 83–105. 7827425 10.1016/0025-5564(94)90025-6

[nph15177-bib-0002] Allen LJ . 2017 A primer on stochastic epidemic models: formulation, numerical simulation, and analysis. Infectious Disease Modelling 2: 128–142. 29928733 10.1016/j.idm.2017.03.001 PMC6002090

[nph15177-bib-0004] Almeida RPP , Nunney L . 2015 How do plant diseases caused by *Xylella fastidiosa* emerge? Plant Disease 99: 1457–1467. 10.1094/PDIS-02-15-0159-FE 30695952

[nph15177-bib-0005] Anderson PK , Cunningham AA , Patel NG , Morales FJ , Epstein PR , Daszak P . 2004 Emerging infectious diseases of plants: pathogen pollution, climate change and agrotechnology drivers. Trends in Ecology & Evolution 19: 535–544. 16701319 10.1016/j.tree.2004.07.021

[nph15177-bib-0006] Ando T . 2011 Predictive Bayesian model selection. American Journal of Mathematical and Management Sciences 31: 13–38.

[nph15177-bib-0007] Aparicio JP , Pascual M . 2007 Building epidemiological models from R0: an implicittreatment of transmission in networks. Proceedings of the Royal Society of London B: Biological Sciences 274: 505–512. 10.1098/rspb.2006.0057 PMC1766386 17476770

[nph15177-bib-0008] Baker R , Bragard C , Caffier D , Candresse T , Gilioli G , Grégoire J‐C , Holb I , Jeger MJ , Karadjova OE , Magnusson C *et al* 2015 Scientific opinion on the risks to plant health posed by *Xylella fastidiosa* in the EU territory, with the identification and evaluation of risk reduction options. EFSA Journal 13: 3989.

[nph15177-bib-0009] Bartoli C , Lamichhane JR , Berge O , Guilbaud C , Varvaro L , Balestra GM , Vinatzer BA , Morris CE . 2015 A framework to gauge the epidemic potential of plant pathogens in environmental reservoirs: the example of kiwifruit canker. Molecular Plant Pathology 16: 137–149. 24986268 10.1111/mpp.12167 PMC6638457

[nph15177-bib-0010] Beckstead J , Meyer SE , Connolly BM , Huck MB , Street LE . 2010 Cheatgrass facilitates spillover of a seed bank pathogen onto native grass species. Journal of Ecology 98: 168–177.

[nph15177-bib-0011] Bosso L , Russo D , Di Febbraro M , Cristinzio G , Zoina A . 2016 Potential distribution of *Xylella fastidiosa* in Italy: a maximum entropy model. Phytopathologia Mediterranea 55: 62–72.

[nph15177-bib-0012] Brauer F , Van den Driessche P , Wu J . 2008 Mathematical epidemiology, Volume 1945 of lecture notes in mathematics. Berlin/Heidelberg, Germany: Springer‐Verlag.

[nph15177-bib-0013] Britton T , House T , Lloyd AL , Mollison D , Riley S , Trapman P . 2015 Five challenges for stochastic epidemic models involving global transmission. Epidemics 10: 54–57. 25843384 10.1016/j.epidem.2014.05.002 PMC4996665

[nph15177-bib-0014] Burdon JJ , Thrall PH . 2008 Pathogen evolution across the agro‐ecological interface: implications for disease management. Evolutionary Applications 1: 57–65. 25567491 10.1111/j.1752-4571.2007.00005.x PMC3352394

[nph15177-bib-0015] Costello CJ , Solow AR . 2003 On the pattern of discovery of introduced species. Proceedings of the National Academy of Sciences, USA 100: 3321–3323. 10.1073/pnas.0636536100 PMC152290 12615995

[nph15177-bib-0016] Cruaud A , Gonzalez A‐A , Godefroid M , Nidelet S , Streito J‐C , Thuillier J‐M , Rossi J‐P , Santoni S , Rasplus J‐Y . 2018 Using insects to detect, monitor and predict the distribution of *Xylella fastidiosa*: a case study in Corsica. bioRxiv doi: 10.1101/241513. 10.1038/s41598-018-33957-z PMC6199265 30353142

[nph15177-bib-0017] Denancé N , Cesbron S , Briand M , Rieux A , Jacques M‐A . 2017a Is *Xylella fastidiosa* really emerging in France? In: Costa J, Koebnik R, eds. 1st Annual Conference of the EuroXanth – COST Action Integrating Science on Xanthomonadaceae for integrated plant disease management in Europe. Dec. 13–15, Coimbra, Portugal: EuroXanth, 7.

[nph15177-bib-0018] Denancé N , Legendre B , Briand M , Olivier V , Boisseson C , Poliakoff F , Jacques M‐A . 2017b Several subspecies and sequence types are associated with the emergence of *Xylella fastidiosa* in natural settings in France. Plant Pathology 66: 1054–1064.

[nph15177-bib-0019] Dobson A . 2004 Population dynamics of pathogens with multiple host species. American Naturalist 164: S64–S78. 10.1086/424681 15540143

[nph15177-bib-0020] Dudas G , Rambaut A . 2014 Phylogenetic analysis of Guinea 2014 EBOV *Ebolavirus* outbreak. PLoS Currents Outbreaks 1: 1–17. 10.1371/currents.outbreaks.84eefe5ce43ec9dc0bf0670f7b8b417d PMC4024086 24860690

[nph15177-bib-0500] European Commission, Directorate‐General for Health and Food Safety . 2017 List of demarcated areas established in the Union territory for the presence of Xylella fastidiosa as referred to in Article 4(1) of Decision (EU) 2015/789, Update 8. Ref. Ares (2017) 3773669‐27/07/2017. https://ec.europa.eu/food/sites/food/files/plant/docs/ph_biosec_legis_list-demarcated-union-territory_en.pdf [accessed 16 April 2018].

[nph15177-bib-0021] Fabre F , Rousseau E , Mailleret L , Moury B . 2012 Durable strategies to deploy plant resistance in agricultural landscapes. New Phytologist 193: 1064–1075. 22260272 10.1111/j.1469-8137.2011.04019.x

[nph15177-bib-0022] Faria NR , Rambaut A , Suchard MA , Baele G , Bedford T , Ward MJ , Tatem AJ , Sousa JD , Arinaminpathy N , Pépin J *et al* 2014 The early spread and epidemic ignition of HIV‐1 in human populations. Science 346: 56–61. 25278604 10.1126/science.1256739 PMC4254776

[nph15177-bib-0023] Fisher MC , Henk DA , Briggs CJ , Brownstein JS , Madoff LC , McCraw SL , Gurr SJ . 2012 Emerging fungal threats to animal, plant and ecosystem health. Nature 484: 186–194. 22498624 10.1038/nature10947 PMC3821985

[nph15177-bib-0024] Gardi C , Koufakis I , Tramontini S , Andueza M , Pautasso M , Stancanelli G , Bau A , Gregoire JC , Bragard C . 2016 Update of a database of host plants of *Xylella fastidiosa*: 20 November 2015. EFSA Journal 14: 4378. 10.2903/j.efsa.2016.4378 PMC13093153 42016881

[nph15177-bib-0025] Gelman A , Carlin JB , Stern HS , Dunson DB , Vehtari A , Rubin DB . 2014 Bayesian data analysis, *3^rd^ edn* Boca Raton, FL, USA: CRC Press.

[nph15177-bib-0026] Gérard PR , Husson C , Pinon J , Frey P . 2006 Comparison of genetic and virulence diversity of *Melampsora larici‐populina* populations on wild and cultivated poplar and influence of the alternate host. Phytopathology 96: 1027–1036. 18944059 10.1094/PHYTO-96-1027

[nph15177-bib-0027] Germain J‐F (2016). Les insectes vecteurs potentiels de *Xylella fastidiosa* en France metropolitaine In Beuste P, Bigel R, Boutte B, Cassignol F, Dours CGO, Ehret P, Gandon M, Gauthier B, Jugnet M‐P, Lacordaire A‐I *et al*, eds. 4e Conférence sur l'Entretien des Jardins, Espaces Végétalisés et Infrastructures, Toulouse, 19 et 20 Octobre 2016. Association Francaise de Protection des Plante, 118–124.

[nph15177-bib-0028] Haydon DT , Cleaveland S , Taylor LH , Laurenson MK . 2002 Identifying reservoirs of infection: a conceptual and practical challenge. Emerging Infectious Diseases 8: 1468–1473. 12498665 10.3201/eid0812.010317 PMC2738515

[nph15177-bib-0029] Heiler KC , Bij de Vaate A , Ekschmitt K , von Oheimb PV , Albrecht C , Wilke T . 2013 Reconstruction of the early invasion history of the quagga mussel (*Dreissena rostriformis bugensis*) in Western Europe. Aquatic Invasions 8: 53–57.

[nph15177-bib-0030] Held L , Hofmann M , Höhle M , Schmid V . 2006 A two‐component model for counts of infectious diseases. Biostatistics 7: 422–437. 16407470 10.1093/biostatistics/kxj016

[nph15177-bib-0031] Holt RD , Dobson AP , Begon M , Bowers RG , Schauber EM . 2003 Parasite establishment in host communities. Ecology Letters 6: 837–842.

[nph15177-bib-0032] Hulme PE . 2009 Trade, transport and trouble: managing invasive species pathways in an era of globalization. Journal of Applied Ecology 46: 10–18.

[nph15177-bib-0033] Jeanmonod D , Schlüssel A , Gamisans J . 2011 Analyse de la ore Corse: aspects biologiques. Candollea 66: 5–25.

[nph15177-bib-0034] Jeger M , Pautasso M , Stack J . 2011 Climate, globalization and trade: impacts on dispersal and invasion of fungal plant pathogens In: Olsen L, Choffnes ER, Relman DA, Pray L, eds. Fungal diseases: an emerging threat to human, animal and plant health. Washington, DC, USA: National Academies Press, 273–296.

[nph15177-bib-0035] Jones DR , Baker RHA . 2007 Introductions of non‐native plant pathogens into Great Britain, 1970–2004. Plant Pathology 56: 891–910.

[nph15177-bib-0036] Karesh WB , Dobson A , Lloyd‐Smith JO , Lubroth J , Dixon MA , Bennett M , Aldrich S , Harrington T , Formenty P , Loh EH *et al* 2012 Ecology of zoonoses: natural and unnatural histories. Lancet 380: 1936–1945. 23200502 10.1016/S0140-6736(12)61678-X PMC7138068

[nph15177-bib-0037] Kass RE , Raftery AE . 1995 Bayes factors. Journal of the American Statistical Association 90: 773–795.

[nph15177-bib-0038] Keeling MJ , Grenfell BT . 2000 Individual‐based perspectives on R0. Journal of Theoretical Biology 203: 51–61. 10677276 10.1006/jtbi.1999.1064

[nph15177-bib-0039] Kleczkowski A , Grenfell BT . 1999 Mean‐field‐type equations for spread of epidemics: the ‘small world’ model. Physica A: Statistical Mechanics and its Applications 274: 355–360.

[nph15177-bib-0040] Le Floc'h E . 1991 Invasive plants of the Mediterranean Basin In: Groves RH, Di Castri F, eds. Biogeography of Mediterranean invasions. Cambridge, UK: Cambridge University Press, 67–80.

[nph15177-bib-0041] Li H , Zhang X , Zheng R , Li X , Elmer WH , Wolfe LM , Li B . 2014 Indirect e_ects of non‐native *Spartina alterniora* and its fungal pathogen (*Fusarium palustre*) on native saltmarsh plants in China. Journal of Ecology 102: 1112–1119.

[nph15177-bib-0042] McCormack RK , Allen LJS . 2006 Stochastic SIS and SIR multihost epidemic models In: Agarwal RP, Perera K, eds. Proceedings of the conference on differential & difference equations and applications. New York, NY, USA: Hindawi Publishing Company, 775–786.

[nph15177-bib-0043] Médail F , Diadema K . 2006 Biodiversité végétale méditerranéenne et anthropisation: approches macro et micro‐régionales. Annales de géographie 651: 618–640.

[nph15177-bib-0044] Mollentze N , Nel LH , Townsend S , le Roux K , Hampson K , Haydon DT , Soubeyrand S . 2014 A Bayesian approach for inferring the dynamics of partially observed endemic infectious diseases from space‐time‐genetic data. Proceedings of the Royal Society B 281: 20133251. 24619442 10.1098/rspb.2013.3251 PMC3973266

[nph15177-bib-0045] Monteil CL , Cai R , Liu H , Mechan Llontop ME , Studholme DJ , Morris CE , Vinatzer BA . 2013 Nonagricultural reservoirs contribute to emergence and evolution of *Pseudomonas syringae* crop pathogens. New Phytologist 199: 800–811. 23692644 10.1111/nph.12316

[nph15177-bib-0046] Morris CE , Kinkel LL , Xiao K , Prior P , Sands DC . 2007 Surprising niche for the plant pathogen *Pseudomonas syringae* . Infection, Genetics and Evolution 7: 84–92. 10.1016/j.meegid.2006.05.002 16807133

[nph15177-bib-0047] Nunes MRT , Palacios G , Faria NR , Sousa EC Jr , Pantoja JA , Rodrigues SG , Carvalho VL , Medeiros DB , Savji N , Baele G *et al* 2014 Air travel is associated with intracontinental spread of dengue virus serotypes 1‐3 in Brazil. PLoS Neglected Tropical Diseases 8: e2769. 24743730 10.1371/journal.pntd.0002769 PMC3990485

[nph15177-bib-0501] Nunes LR , Rosato YB , Muto NH , Yanai GM , da Silva VS , Leite DB , Gonçalves ER , de Souza AA , Coletta‐Filho HD , Machado MA *et al* 2003 Microarray analyses of *Xylella fastidiosa* provide evidence of coordinated transcription control of laterally transferred elements. Genome Research 13: 570–578. 12670998 10.1101/gr.930803 PMC430171

[nph15177-bib-0502] Nunney L , Yuan X , Bromley RE , Hartung J , Montero‐Astúa M , Moreira L , Ortiz B , Stouthamer R . 2010 Population genomic analysis of a bacterial plant pathogen: novel insight into the origins of Pierce’s disease of grapevine in the U.S. PLoS ONE 55: e15488. 10.1371/journal.pone.0015488 PMC2982844 21103383

[nph15177-bib-0048] Olsen L , Choffnes ER , Relman DA , Pray L . 2011 Workshop overview In: Olsen L, Choffnes ER, Relman DA, Pray L, eds. Fungal diseases: an emerging threat to human, animal and plant health. Washington, DC, USA: National Academies Press, 1–99. 22259817

[nph15177-bib-0049] Potter C , Harwood T , Knight J , Tomlinson I . 2011 Learning from history, predicting the future: the UK Dutch elm disease outbreak in relation to contemporary tree disease threats. Philosophical Transactions of the Royal Society of London B: Biological Sciences 366: 1966–1974. 21624917 10.1098/rstb.2010.0395 PMC3130388

[nph15177-bib-0050] Preston CD , Pearman DA , Hall AR . 2004 Archaeophytes in Britain. Botanical Journal of the Linnean Society 145: 257–294.

[nph15177-bib-0051] Purcell A . 2013 Paradigms: examples from the bacterium *Xylella fastidiosa* . Annual Review of Phytopathology 51: 339–356. 10.1146/annurev-phyto-082712-102325 23682911

[nph15177-bib-0052] Redak RA , Purcell AH , Lopes JR , Blua MJ , Mizell Iii RF , Andersen PC . 2004 The biology of xylem UID‐feeding insect vectors of *Xylella fastidiosa* and their relation to disease epidemiology. Annual Reviews in Entomology 49: 243–270. 10.1146/annurev.ento.49.061802.123403 14651464

[nph15177-bib-0053] Saponari M , Loconsole G , Cornara D , Yokomi RK , Stradis AD , Boscia D , Bosco D , Martelli GP , Krugner R , Porcelli F . 2014 Infectivity and transmission of *Xylella fastidiosa* by *Philaenus spumarius* (Hemiptera: Aphrophoridae) in Apulia, Italy. Journal of Economic Entomology 107: 1316–1319. 25195417 10.1603/ec14142

[nph15177-bib-0054] Soubeyrand S , Garreta V , Monteil C , Suffert F , Goyeau H , Berder J , Moinard J , Fournier E , Tharreau D , Morris CE *et al* 2017 Testing differences between pathogen compositions with small samples and sparse data. Phytopathology 107: 1199–1208. 28677479 10.1094/PHYTO-02-17-0070-FI

[nph15177-bib-0055] Soubeyrand S , Laine A , Hanski I , Penttinen A . 2009 Spatio‐temporal structure of host‐pathogen interactions in a metapopulation. American Naturalist 174: 308–320. 10.1086/603624 19627233

[nph15177-bib-0056] Soubeyrand S , Roques L . 2014 Parameter estimation for reaction‐diffusion models of biological invasions. Population Ecology 56: 427–434.

[nph15177-bib-0057] Spiegelhalter DJ , Best NG , Carlin BP , Van Der Linde A . 2002 Bayesian measures of model complexity and fit. Journal of the Royal Statistical Society B 64: 583–639.

[nph15177-bib-0058] Stakman EC (1919). The black stem rust and the barberry In: Houston DF, Ousley C, Harrison FR, Taylor AE, Williams WM, Taylor HC, Reese RM, Gladmon PL, Lamson‐Scribner F, Smith H *et al*, eds. Yearbook of the United States Department of Agriculture 1918. Washington, DC, USA: Washington Government Printing Office, 75–100.

[nph15177-bib-0059] Tatem AJ , Rogers DJ , Hay S . 2006 Global transport networks and infectious disease spread. Advances in Parasitology 62: 293–343. 16647974 10.1016/S0065-308X(05)62009-X PMC3145127

[nph15177-bib-0060] Unkel S , Farrington C , Garthwaite PH , Robertson C , Andrews N . 2012 Statistical methods for the prospective detection of infectious disease outbreaks: a review. Journal of the Royal Statistical Society: Series A (Statistics in Society) 175: 49–82.

[nph15177-bib-0061] Viana M , Mancy R , Biek R , Cleaveland S , Cross PC , Lloyd‐Smith JO , Haydon DT . 2014 Assembling evidence for identifying reservoirs of infection. Trends in Ecology & Evolution 29: 270–279. 24726345 10.1016/j.tree.2014.03.002 PMC4007595

[nph15177-bib-0062] Waage JK , Woodhall JW , Bishop SJ , Smith JJ , Jones DR , Spence NJ . 2008 Patterns of plant pest introductions in Europe and Africa. Agricultural Systems 99: 1–5.

[nph15177-bib-0063] White SM , Bullock JM , Hooftman DA , Chapman DS . 2017 Modelling the spread and control of *Xylella fastidiosa* in the early stages of invasion in Apulia, Italy. Biological Invasions 19: 1825–1837. 10.1007/s10530-017-1393-5 PMC6979717 32025190

[nph15177-bib-0064] Woolhouse M , Gaunt E . 2007 Ecological origins of novel human pathogens. Critical Reviews in Microbiology 33: 231–242. 18033594 10.1080/10408410701647560

