# Supplementary material for: Inferring pathogen dynamics from temporal count data: the emergence of Xylella fastidiosa in France is probably not recent
Source: New Phytol. 2018 Apr 24;219(2):824–36. doi: 10.1111/nph.15177 (PMC6032966; doi:10.1111/nph.15177)
Supplement: Supplementary file 1 — Fig. S1 Locations of Xylella fastidiosa positive and negative samples in Corsica, France, between July 2015 and December 2016. Fig. S2 Prior and posterior distributions of I 0 under model M8 when I 0 is not fixed. Fig. S3 Posterior means and quantiles of parameters of model M8 obtained for various values of I 0. Fig. S4 Posterior medians, 0.025 quantiles and 0.975 quantiles of the past proportions of infected hosts under models M3 and M8. Fig. S5 Marginal posterior distributions of the parameters of model M3. Fig. S6 Marginal posterior distributions of the parameters of model M8. Fig. S7 Posterior medians, 0.025 quantiles and 0.975 quantiles of the future proportions of infected hosts under model M3 and different surveillance scenarios. Fig. S8 Posterior medians, 0.025 quantiles and 0.975 quantiles of the future proportions of infected hosts under model M8 and different surveillance scenarios. Table S1 Monthly surveillance data in South Corsica Table S2 Specifications of the hidden compartment and the preference in sampling for models M1,…,M8, with mathematical expressions of the function g Table S3 Prior distributions of parameters Table S4 Posterior means, medians, 0.025 quantiles and 0.975 quantiles of parameters of models M3 and M8 Table S5 Posterior means, medians, 0.025 quantiles and 0.975 quantiles of the introduction year and the number/proportion of infected hosts in December 2016 under models M3 and M8 Notes S1 List of plant genera sampled in South Corsica from July 2015 to December 2016. Notes S2 Detailed model description. Notes S3 Impact of the choice of the number I 0 of introduced infected hosts on the estimation of the other model parameters. [file NPH-219-824-s001.pdf]

Supplementary Material

Inferring pathogen dynamics from temporal count data: The emergence of *Xylella fastidiosa* in France is probably not recent

Soubeyrand S., de Jerphanion P., Martin O., Saussac M.,
Manceau C., Hendrikx P., Lannou C.

Acceptance date: 16 March 2018

Supplementary Notes S1: List of plant genera sampled in South Corsica from July 2015 to December 2016

Abelia Acacia Acer Achillea Aesculus Albizia Alnus Aloysia Ampelopsis Anisodontea Anthyllis Aralia Arbutus Artemisia Asparagus Asperula Aster Astragalus Bambusa Betula Bougainvillea Brassica Buddleja Buxus Calicotome Callistemon Camellia Campsis Carduus Cassia Castanea Catharanthus Celtis Centranthus Cercis Choisya Cistus Citrus Clematis Clinopodium Convolvulus Coronilla Corylus Cotoneaster Crataegus Cucurbita Cupressus Cynareae Cynodon Cyperus Cytisophyllum Cytisus Daphne Deutzia Digitaria Dittrichia Elaeagnus Elytrigia Encelia Erica Erigeron Escallonia Eucalyptus Eugenia Euonymus Euryops Fagus Feijoa Ficus Foeniculum Fragaria Fraxinus Genista Geranium Ginkgo Glebionis Grevillea Hebe Hedera Helianthus Helichrysum Helleborus Hibiscus Hydrangea Hypericum Ilex Ipomoea Jacaranda Jacobaea Juglans Juniperus Lagerstroemia Lantana Lathyrus Laurocerasus Laurus Lavandula Leucophyllum Ligustrum Liquidambar Lonicera Lupinus Lycianthes Magnolia Malus Medicago Mentha Metrosideros Morus Myrtus Nerium Olea Osmanthus Osyris Oxalis Parthenocissus Pelargonium Persea Phagnalon Philadelphus Phillyrea Photinia Phytolacca Pistacia Pittosporum Plantago Platanus Plumbago Poaceae Polygala Populus Portulaca Prunus Pteridium Punica Pyrus Quercus Raphanus Rhamnus Rhododendron Ricinus Robinia Rosa Rosmarinus Rubus Ruscus Rutaceae Salicornia Salix Salvia Sambucus Schinus Scrophularia Silybum Smilax Solandra Solanum Sorbus Spartium Stachys Syringa Tamarix Teucrium Thymus Tilia Trifolium Ulmus Umbellularia Urtica Vaccinium Veronica Viburnum Vicia Vinca Vitis Washingtonia Weigela Westringia Wisteria Yucca

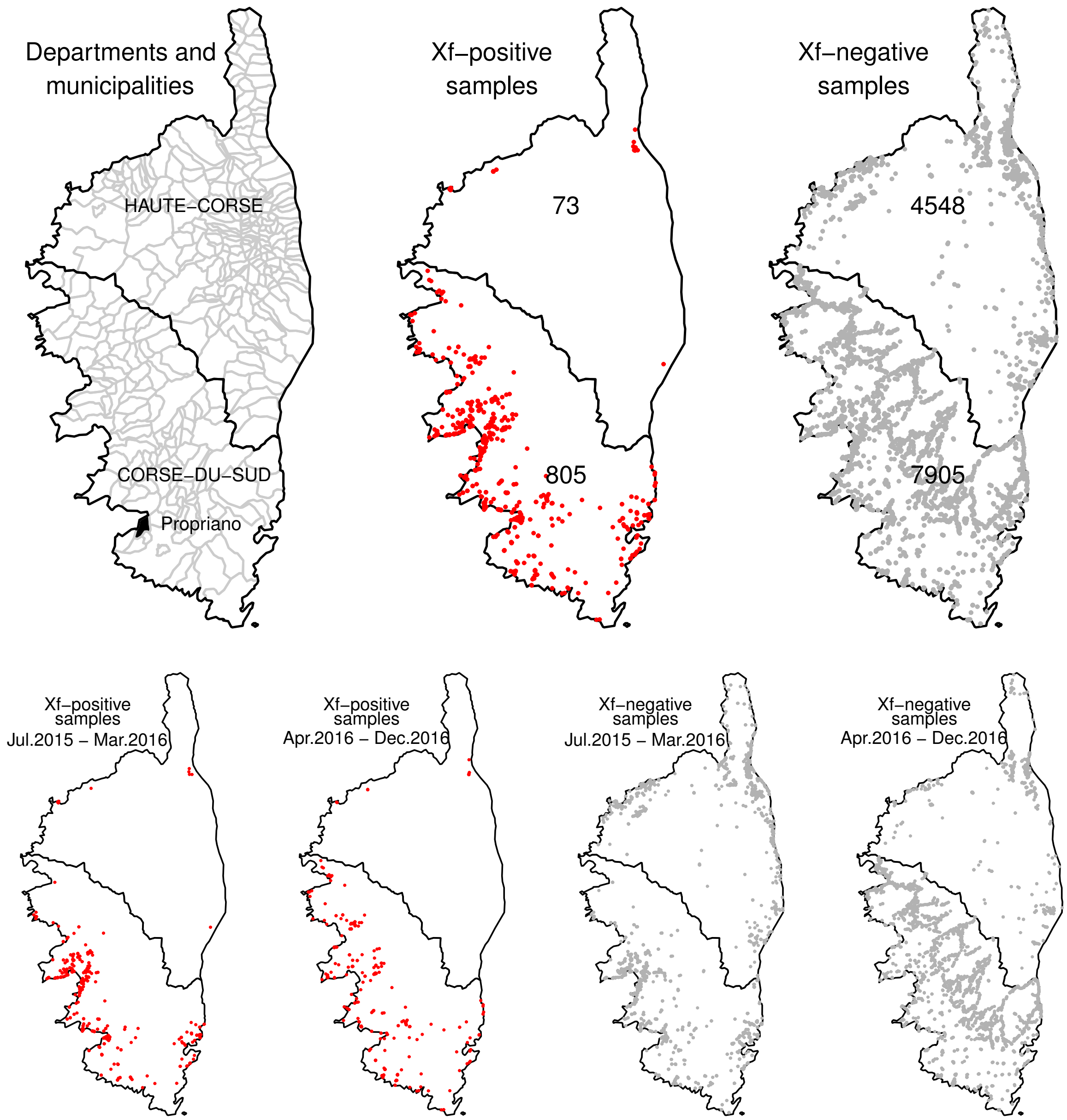

Figure S1: Top left: Borders of the French departments (Haute-Corse and Corse-du-Sud) partitioning Corsica (black lines), borders of Corsican municipalities (grey lines) and location of Propriano (black shape), where the first plant positive to *X. fastidiosa* (Xf) was detected in July 2015. Top center: Locations of Xf–positive samples collected between July 2015 and December 2016, together with the count of such samples in each department. Top right: Locations of Xf–negative samples collected between July 2015 and December 2016, together with the count of such samples in each department. Note that positive cases are mostly near the coastline where the bacteria faces mild winters, whereas the central Corsica is a higher-altitude (and colder) region (especially in winter). Bottom: Locations of Xf–positive and Xf–negative samples over the first half and the second half of the data period, namely from July 2015 to March 2016 and from April 2016 to December 2016, respectively.

Year-Month	N_{obs}	I_{obs}	$N^{\dagger}_{obs}$	$I^{\dagger}_{obs}$	$N^{\emptyset}_{obs}$	$I^{\emptyset}_{obs}$	CC(species)	CC(municipalities)
2015-07	107	28	47	27	60	1	29	17
2015-08	418	126	288	112	130	14	91	30
2015-09	746	109	538	101	208	8	176	45
2015-10	930	104	616	89	314	15	234	51
2015-11	507	63	260	51	247	12	257	56
2015-12	240	17	169	15	71	2	269	60
2016-01	158	23	111	21	47	2	269	60
2016-02	167	9	141	8	26	1	271	60
2016-03	72	7	63	6	9	1	272	60
2016-04	384	24	302	20	82	4	285	62
2016-05	433	51	397	51	36	0	296	67
2016-06	492	84	383	83	109	1	307	76
2016-07	343	38	269	37	74	1	314	83
2016-08	366	23	227	10	139	13	326	89
2016-09	402	10	347	7	55	3	341	93
2016-10	880	23	843	22	37	1	358	105
2016-11	1087	52	1053	52	34	0	368	120
2016-12	170	13	111	4	59	9	371	120

Table S1: Monthly surveillance data in South Corsica. $N_{obs} = N^{\dagger}_{obs} + N^{\emptyset}_{obs}$: number of sampled hosts; $N^{\dagger}_{obs}$: number of sampled symptomatic hosts; $N^{\emptyset}_{obs}$: number of sampled asymptomatic hosts; $I_{obs} = I^{\dagger}_{obs} + I^{\emptyset}_{obs}$: number of sampled infected hosts; $I^{\dagger}_{obs}$: number of sampled infected symptomatic hosts; $I^{\emptyset}_{obs}$: number of sampled infected asymptomatic hosts; CC: cumulated count (of species or municipalities).

Supplementary Notes S2: Detailed model description

Model skeleton

Time is discrete and regular, and takes values in the set of integers $\mathbb{Z}$ (in our application, the time unit is the month). The unobserved process Y represents the real epidemic and is split into 4 components:

$$Y(t) = \begin{pmatrix} S_{\mathrm{O}}(t) \\ S_{\mathrm{H}}(t) \\ I_{\mathrm{O}}(t) \\ I_{\mathrm{H}}(t) \end{pmatrix},$$

where

- $S_{\mathrm{O}}(t)$ is the number of susceptible hosts at time t in the observable compartment,
- $S_{\mathrm{H}}(t)$ is the number of susceptible hosts at time t in the hidden compartment,
- $I_{\mathrm{O}}(t)$ is the number of infected hosts at time t in the observable compartment,
- $I_{\mathrm{H}}(t)$ is the number of infected hosts at time t in the hidden compartment.

Thereafter, indices O and H are used to distinguish variables concerning the observable and hidden compartments, respectively.

The epidemic is observed via the surveillance of symptomatic and asymptomatic hosts in the observable compartment. In addition, hosts which are observed as infected are destroyed. Thus, the observation process is a so-called control process, which changes the course of the epidemic (the aim of the control may be to eradicate or slow down the epidemic). The control process consists of a deterministic component C_N providing the numbers of symptomatic and asymptomatic sampled hosts at time t, and a stochastic component C_I providing the numbers of symptomatic and asymptomatic infected hosts among the sampled hosts:

$$C_N(t) = \begin{pmatrix} N^{\dagger}_{\mathrm{obs}}(t) \\ N^{\emptyset}_{\mathrm{obs}}(t) \end{pmatrix}, \qquad C_I(t) = \begin{pmatrix} I^{\dagger}_{\mathrm{obs}}(t) \\ I^{\emptyset}_{\mathrm{obs}}(t) \end{pmatrix},$$

where

- $N^{\dagger}_{\mathrm{obs}}(t)$ is the number of symptomatic hosts sampled at time t in the observable compartment,
- $N^{\emptyset}_{\mathrm{obs}}(t)$ is the number of asymptomatic hosts sampled at time t in the observable compartment,
- $I^{\dagger}_{\mathrm{obs}}(t)$ is the number of infected hosts at time t among the $N^{\dagger}_{\mathrm{obs}}(t)$ sampled symptomatic hosts,
- $I^{\emptyset}_{\mathrm{obs}}(t)$ is the number of infected hosts at time t among the $N^{\emptyset}_{\mathrm{obs}}(t)$ sampled asymptomatic hosts.

Thereafter, symbols $\dagger$ and $\emptyset$ are used to distinguish variables concerning symptomatic and asymptomatic hosts, respectively,

The process C_N, which represents the sampling effort, is supposed to be known. When no host is observed at time t, the two components of C_N are equal to zero. The processes Y and C_I are updated as follows:

$$\begin{cases} Y(t) & = F_\theta(Y(t-1), \ldots, Y(t-K); C_I(t-1)) \\ C_I(t) & = G_\theta(Y(t); C_N(t)) \end{cases}$$

where the new state of Y depends on the past states of Y (up to time $t-K$, $K \in \mathbb{N}^*$) and the previous observation of infected hosts, the new state of C_I depends on the new state of the epidemic and the (deterministic) numbers of sampled hosts, and the functions F_θ and G_θ parameterized by θ govern these dependencies.

The two following sections describe how we specified F_θ (i.e. the controlled epidemic process) and G_θ (i.e. the observation process). Then, we will specify the prior distributions of model parameters.

Model of the controlled epidemic process

Before the time of introduction of the disease, say t_0, the process Y is constant and equal to:

$$Y(t) = \begin{pmatrix} S_{\mathrm{O}}(t) \\ S_{\mathrm{H}}(t) \\ I_{\mathrm{O}}(t) \\ I_{\mathrm{H}}(t) \end{pmatrix} = \begin{pmatrix} \lfloor \phi N_0 \rceil \\ \lfloor (1-\phi) N_0 \rceil \\ 0 \\ 0 \end{pmatrix}, \text{ for all } t < t_0,$$

where N_0 is the initial host population size, ϕ is the initial proportion of the host population corresponding to the observable compartment, and $\lfloor \cdot \rceil$ is the rounding operator.

The epidemic is initiated at time t_0 by distributing I_0 infected hosts in the observable and hidden compartments, with the proportions ϕ and $(1-\phi)$, respectively:

$$Y(t_0) = \begin{pmatrix} \lfloor \phi N_0 \rceil - \lfloor \phi I_0 \rceil \\ \lfloor (1-\phi) N_0 \rceil - \lfloor (1-\phi) I_0 \rceil \\ \lfloor \phi I_0 \rceil \\ \lfloor (1-\phi) I_0 \rceil \end{pmatrix}.$$

At time $t > t_0$, new infections in the observable and hidden compartments, say $I_{\mathrm{O}}^*(t)$ and $I_{\mathrm{H}}^*(t)$ respectively, occur as a function of past prevalences:

$$I_{\mathrm{O}}^*(t) = \left\lfloor \min\left\{1, \sum_{k=1}^{K} w_k \frac{I(t-k)}{N(t-k)}\right\} S_{\mathrm{O}}(t-1) \right\rceil \tag{1}$$

$$I_{\mathrm{H}}^*(t) = \left\lfloor \min\left\{1, \sum_{k=1}^{K} w_k \frac{I(t-k)}{N(t-k)}\right\} S_{\mathrm{H}}(t-1) \right\rceil \tag{2}$$

where $N(t-k)$ is the total number of hosts at time $t-k$ (i.e. the sum of the 4 components of $Y(t-k)$), $I(t-k) = I_{\mathrm{O}}(t-k) + I_{\mathrm{H}}(t-k)$ is the total number of infected hosts at time $t-k$, and $w_k \geq 0$ is a weight measuring the contribution of the disease prevalence at time $t-k$ to new infections at time t. Thus, $w_k I(t-k)/N(t-k)$ gives the potential of infection of hosts infected at time $t-k$ over hosts susceptible at time $t-1$, and $\min\left\{1, \sum_{k=1}^{K} w_k \frac{I(t-k)}{N(t-k)}\right\}$ gives the probability of infection at time t of hosts that were susceptible at time $t-1$. In this model, any infected host contributes to the new infections in both the observable and hidden compartments, irrespective of the compartment it belongs to. Equations (1–2), without the rounding and minimum operators, can be viewed as the

auto-regressive terms appearing in the discrete-time renewal equation[1], which is often used to build temporal models of disease incidence, like in Champredon et al. (2017).

In addition, at time $t > t_0$,

1. infected hosts observed at time $t-1$ are removed from the infected hosts in the observable compartment and replaced by resistant hosts[2], in the aim of enhancing the spread of the disease;
2. the other infected hosts at time $t-1$ are affected by a mortality rate ρ, irrespective of the compartments they belong to, and they are replaced by susceptible hosts[3].

These assumptions and Equations (1–2) lead to:

$$Y(t) = \begin{pmatrix} S_{\mathrm{O}}(t-1) \\ S_{\mathrm{H}}(t-1) \\ I_{\mathrm{O}}(t-1) \\ I_{\mathrm{H}}(t-1) \end{pmatrix} + \begin{pmatrix} \lfloor \rho\{I_{\mathrm{O}}(t-1) - I_{\mathrm{obs}}(t-1)\} \rceil - I^*_{\mathrm{O}}(t) \\ \lfloor \rho I_{\mathrm{H}}(t-1) \rceil - I^*_{\mathrm{H}}(t) \\ -I_{\mathrm{obs}}(t-1) - \lfloor \rho\{I_{\mathrm{O}}(t-1) - I_{\mathrm{obs}}(t-1)\} \rceil + I^*_{\mathrm{O}}(t) \\ -\lfloor \rho I_{\mathrm{H}}(t-1) \rceil + I^*_{\mathrm{H}}(t) \end{pmatrix}, \text{ for all } t > t_0, \quad (3)$$

where $I_{\mathrm{obs}}(t-1) = I^{\dagger}_{\mathrm{obs}}(t-1) + I^{\emptyset}_{\mathrm{obs}}(t-1)$ is the number of (symptomatic and asymptomatic) infected hosts detected at time $t-1$, and the rounding operator allows us to obtain a vector of integers for $Y(t)$. The number of removed hosts in our SIR model is defined in Footnote 2.

Model of the observation process

We modeled the numbers $I^{\dagger}_{\mathrm{obs}}(t)$ and $I^{\emptyset}_{\mathrm{obs}}(t)$ of symptomatic and asymptomatic observed infected hosts at times t satisfying $N^{\dagger}_{\mathrm{obs}}(t) > 0$ and $N^{\emptyset}_{\mathrm{obs}}(t) > 0$ with independent hypergeometric distributions taking into account the effects of false-negative in the diagnostic test:

$$I^{\dagger}_{\mathrm{obs}}(t) \sim \mathcal{H}\left(\left\lfloor (1-\epsilon) I^{\dagger}_{\mathrm{O}}(t) \right\rceil, S^{\dagger}_{\mathrm{O}}(t) + \left\lfloor \epsilon I^{\dagger}_{\mathrm{O}}(t) \right\rceil, N^{\dagger}_{\mathrm{obs}}(t)\right) \quad (4)$$

$$I^{\emptyset}_{\mathrm{obs}}(t) \sim \mathcal{H}\left(\left\lfloor (1-\epsilon) I^{\emptyset}_{\mathrm{O}}(t) \right\rceil, S^{\emptyset}_{\mathrm{O}}(t) + \left\lfloor \epsilon I^{\emptyset}_{\mathrm{O}}(t) \right\rceil, N^{\emptyset}_{\mathrm{obs}}(t)\right), \quad (5)$$

where

- $I^{\dagger}_{\mathrm{O}}(t)$ is the number of infected symptomatic hosts at time t in the observable compartment, which is assumed to be a fixed proportion $\gamma_1 \in [0,1]$ of $I_{\mathrm{O}}(t)$:

$$I^{\dagger}_{\mathrm{O}}(t) = \gamma_1 I_{\mathrm{O}}(t);$$

- $I^{\emptyset}_{\mathrm{O}}(t)$ is the number of infected asymptomatic hosts at time t in the observable compartment

[1] *Definition: Let $\{a_n\}$ and $\{b_t\}$ be two sequences of real values indexed by $n \in \mathbb{N}$ and $t \in \mathbb{Z}$, respectively. The sequence $\{u_t\}$ of real values indexed by $t \in \mathbb{Z}$ is said to satisfy a discrete-time renewal equation when $u_t = b_t + \sum_{k\in\mathbb{N}} a_k u_{t-k}$ (Feller, 1957, p. 290).*

[2] The *removed* component of our SIR model is incremented by these resistant hosts: $R(t) = R(t-1) + I_{\mathrm{obs}}(t-1)$, where $R(t)$ is the number of removed hosts at time t and is equal to zero before t_0.

[3] In an older version of the model, we included a rate of resistance when infected hosts, which are not known to be infected, die. The rate of resistance is the rate at which such hosts are replaced by resistant hosts. However, the inference was more complicated and data did not seem to provide information about this parameter.

and satisfies:

$$I_{\mathrm{O}}^{\emptyset}(t) = I_{\mathrm{O}}(t) - I_{\mathrm{O}}^{\dagger}(t) = (1-\gamma_1)I_{\mathrm{O}}(t);$$

- $S_{\mathrm{O}}^{\dagger}(t)$ is the number of susceptible symptomatic hosts at time t in the observable compartment that are considered as *at-risk* (i.e. hosts whose propensity to be sampled is high, despite their healthy status); this number is assumed to be a time-varying proportion $g(t)\gamma_2$ of $S_{\mathrm{O}}(t)$:

$$S_{\mathrm{O}}^{\dagger}(t) = g(t)\gamma_2 S_{\mathrm{O}}(t),$$

  where $\gamma_2 \in [0,1]$ is the fixed proportion of symptomatic hosts among the at-risk susceptible hosts in the observable compartment, and $g(t)$ is the time-varying proportion of at-risk hosts among the susceptible hosts in the observable compartment;
- $S_{\mathrm{O}}^{\emptyset}(t)$ is the number of susceptible asymptomatic hosts at time t in the observable compartment that are considered as *at-risk*, and satisfies:

$$S_{\mathrm{O}}^{\emptyset}(t) = g(t)(1-\gamma_2)S_{\mathrm{O}}(t);$$

- ϵ is the rate of false-negatives: the fraction ϵ of infected hosts cannot be observed as positive cases because of diagnostic test issues, and are accounted for in the negative cases in Equations (4-5).

Note that our model assumes that the rate of false-positives is zero, and that all infected hosts in the observable compartment are *at-risk* (i.e. all infected hosts in the observable compartment have a high propensity to be sampled). Note also that the rounding operator in the hypergeometric distributions is applied to get integers for the parameters.

Competing models and prior distributions

We built eight competing models, by considering several options for the initial proportion ϕ of the host population that is observable, and several options for the g function providing the time-varying proportion of at-risk hosts among the susceptible hosts in the observable compartment. These models are labelled $\mathcal{M}_1, \ldots, \mathcal{M}_8$ and are classified in Table S2.

Model parameters are listed in Table S3, which also provides their prior distributions. Below we justify the choices of priors. For the introduction date t_0, we chose a vague uniform prior over a period of 50 years (i.e. 600 months) before the first detection that occurred at time $t = 0$ (i.e. July 2015).

Specifying the prior for the initial population size N_0 is tricky and requires to define the host unit. We considered the host-unit to be an area that makes sense regarding the type of data under consideration. Samples are collected from individual plants, but generally represent a set of neighbor plants. The median distance between the locations of samples being 9 meters, we considered that an host unit is an area of about $\pi(9/2)^2 \approx 64$ square meters. Using Corine Land Cover data, the total area potentially covered with plants represents about 3500km^2 in South Corsica, 10% of which was arbitrarily considered as covered by potential host plants of *Xylella fastidiosa*. Thus, we considered that the initial population of host plants consisted of approximately $0.1 \times (3500 \times 10^6)/(\pi(9/2)^2) \approx$

5.5×10^6 host units. Based on this rough evaluation of N_0, we set a relatively vague prior for this parameter, namely, a log-normal distribution with mean and standard deviation parameters equal to $\log(5 \times 10^6)$ and 0.5, respectively, which lead to quantiles of order 0.025 and 0.975 equal to 1.9 million and 13.3 million.

The prior distribution for I_0 was supposed to be Dirac(10) (i.e. I_0 can only take the value 10), which means that 10 infected host units were introduced at t_0. Obviously, this number has a strong influence on the introduction date: a larger I_0 would lead to a more recent introduction date t_0. The dependence between I_0 and t_0 yields an identifiability issue in the estimation algorithm, and led us to fix the value I_0. The value 10 was considered as a plausible number (it might correspond, for instance, to a non negligible volume of infected plants imported by a nursery).

The prior for the mortality rate ρ is a log-normal distribution with mean and standard deviation parameters equal to $\log(0.02)$ and 0.5, respectively, whose quantiles of order 0.025 and 0.975 are 0.0075 and 0.0533. If $\rho = 0.0075$, 50% of infected hosts die in the first 7.7 years of their infection. If $\rho = 0.0533$, 50% of infected hosts die in the first year of their infection. Thus, a priori, the mortality rate can take diverse values corresponding to significantly different mortality dynamics.

Because of some identifiability issues in the estimation algorithm and to allow an eventual annual periodicity to be inferred, the weights w_k were all assumed to be equal to 0, except w_{12}, for which we specified a vague uniform prior over $[0, 10]$. In the main text, w_{12} is denoted by w.

A priori, the false-negative rate ϵ has a uniform prior over $[0, 0.2]$ and is therefore assumed to be relatively low, but it can take non-negligible values.

For the proportions ϕ, γ_1, γ_2, β_1 and β_2, we chose vague uniform priors over $[0, 1]$, except mentioned otherwise in Table S3 to satisfy the specification of each competing model. For instance, for models $\mathcal{M}_1, \mathcal{M}_2, \mathcal{M}_3$ without hidden compartment, ϕ can only be equal to 1, and for models $\mathcal{M}_7$ (with an *a priori* small hidden compartment) and $\mathcal{M}_8$ (with an *a priori* large hidden compartment), ϕ has a beta distribution with parameter vectors equal to (4,1) and (1,4), respectively.

	**Hidden compartment**	
	None	Fraction of the whole population
**Preference in sampling**	$\phi = 1$	$\phi \in [0, 1]$
None<br>$g(t) = 1$	$\mathcal{M}_1$	$\mathcal{M}_4$
At-risk, constant<br>$g(t) = \beta_1 \in [0, 1]$	$\mathcal{M}_2$	$\mathcal{M}_5$
At-risk, linearly varying<br>$g(t) = \beta_1 + (\beta_2 - \beta_1)\frac{t - t_{\text{obs}}^{\min}}{t_{\text{obs}}^{\max} - t_{\text{obs}}^{\min}}$	$\mathcal{M}_3$	$\mathcal{M}_6, \mathcal{M}_7, \mathcal{M}_8$

Table S2: Specifications of the hidden compartment and the preference in sampling for models $\mathcal{M}_1, \ldots, \mathcal{M}_8$. Note that models $\mathcal{M}_6$, $\mathcal{M}_7$ and $\mathcal{M}_8$ have different prior distributions for the parameter ϕ; see Table S3. $t_{\text{obs}}^{\min}$ and $t_{\text{obs}}^{\max}$ are, respectively, the first and last times of observation.

Parameter	Signification	Model	Prior distribution
t_0	Introduction date	All	Uniform($\{-600, \ldots, -1\}$)
N_0	Initial population size	All	Lognormal($\log(5 \times 10^6), 0.5$)
I_0	Number of infecteds at t_0	All	Dirac(10)
ϕ	Initial proportion of the host population that is observable	$\mathcal{M}_1, \mathcal{M}_2, \mathcal{M}_3$	Dirac(1)
		$\mathcal{M}_4, \mathcal{M}_5, \mathcal{M}_6$	Uniform($[0, 1]$)
		$\mathcal{M}_7$	Beta(4,1)
		$\mathcal{M}_8$	Beta(1,4)
ρ	Mortality rate	All	Lognormal($\log(0.02), 0.5$)
w_k	Weight	All	Dirac(0) for $k \neq 12$
		All	Uniform($[0, 10]$) for $k = K = 12$
γ_1	Proportion of symptomatic hosts among infected hosts in the observable compartment	All	Uniform($[0, 1]$)
γ_2	Proportion of symptomatic hosts among at-risk susceptible hosts in the observable compartment	All	Uniform($[0, 1]$)
β_1	Proportion of at-risk susceptible hosts at $t_{\text{obs}}^{\text{min}}$	$\mathcal{M}_1, \mathcal{M}_4$	Dirac(1)
		$\mathcal{M}_2, \mathcal{M}_3, \mathcal{M}_5, \ldots, \mathcal{M}_8$	Uniform($[0, 1]$)
β_2	Proportion of at-risk susceptible hosts at $t_{\text{obs}}^{\text{max}}$	$\mathcal{M}_1, \mathcal{M}_4$	Dirac(1)
		$\mathcal{M}_2, \mathcal{M}_5$	Dirac(β_1)
		$\mathcal{M}_3, \mathcal{M}_6, \mathcal{M}_7, \mathcal{M}_8$	Uniform($[0, 1]$)
ϵ	False-positive rate	All	Uniform($[0, 0.2]$)

Table S3: Prior distributions of parameters. Note that, in the main text, w_{12} is denoted by w.

References

Champredon, D., M. Li, B. M. Bolker, and J. Dushoff (2017). Two approaches to forecast Ebola synthetic epidemics. *Epidemics*, in press.

Feller, W. (1957). *An introduction to probability theory and its applications: volume I, 2nd Ed.* New York: John Wiley & Sons.

Supplementary Notes S3: Impact of the choice of the number I_0 of introduced infected hosts

As indicated in the main text, the number I_0 of introduced infected hosts at t_0, was set at a fixed value in all models because of some identifiability issues. We set $I_0 = 10$, which amounts to assume that the epidemic began with the introduction of a small batch of infected plants and that subsequent introductions did not significantly impact the overall curse of the epidemic.

Here, we illustrate the identifiability issue encountered when I_0 is not fixed, and we investigate the impact of the value chosen for I_0 on the estimation of other parameters. For the sake of example, we used model $\mathcal{M}_8$ that includes an a priori large hidden compartment and a varying preference in sampling at-risk hosts.

Figure S2 provides an example of posterior distribution obtained for I_0 when this parameter is not fixed at 10 but a priori distributed under a log-normal distribution with mean parameter $\log 10$ and standard deviation 1. Despite a prior whose mass is mostly concentrated over values lower than 100, the MCMC chains made excursions to values larger than 1000.

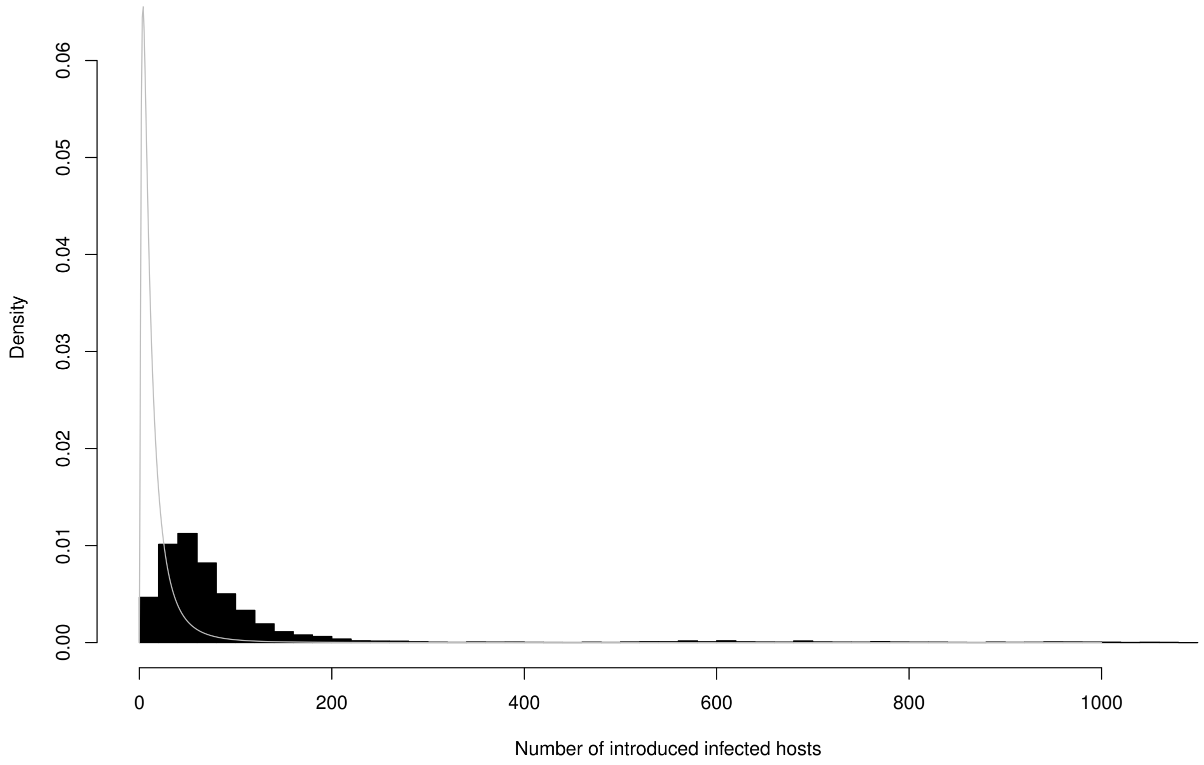

Figure S2: Prior (curve) and posterior (histogram) distributions of I_0 under model $\mathcal{M}_8$ when I_0 is not fixed at 10 but a priori distributed under a log-normal distribution with mean parameter $\log 10$ and standard deviation 1.

We performed additional estimations of parameters of model $\mathcal{M}_8$ by varying the fixed value of I_0. Figure S3 gives, for each model parameter, its posterior mean and quantiles of order 0.025 and 0.975 when I_0 varies between 1 and 100. The value $I_0 = 10$ is indicated by the vertical dashed red line. Figure S3 also shows the posterior mean and quantiles of the log-likelihood that is a measure of the goodness-of-fit of the model. Large differences in posterior means and quantiles are observed for low values of I_0, namely $I_0 = 1$ or $I_0 = 5$, but such low values lead to rather low log-likelihoods. In contrast, larger values of I_0 led to roughly similar results and log-likelihoods. It has however to be noted that the value $I_0 = 10$ generates an introduction date that tends to be more recent than larger values. This is somehow counterintuitive but this result is due, in particular, to the larger estimate obtained for the infection strength when $I_0 = 10$.

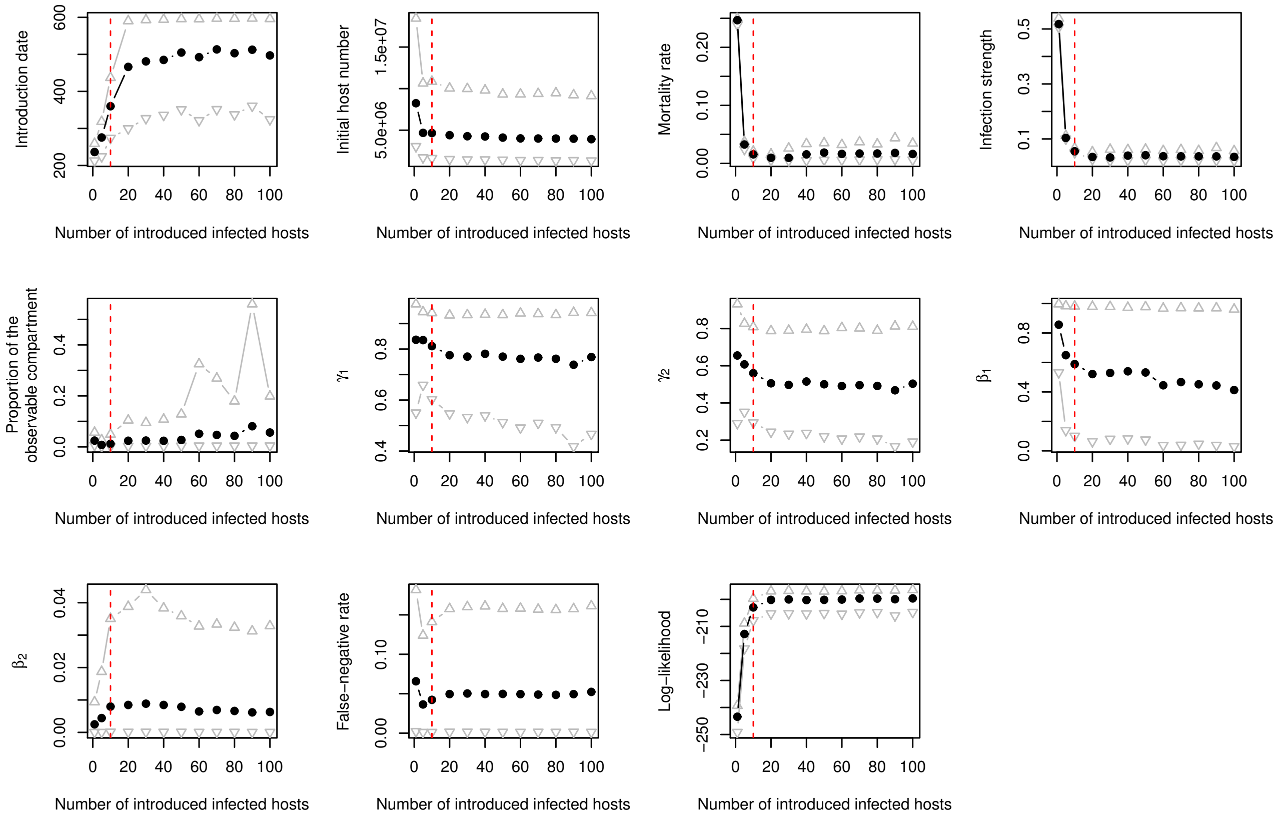

Figure S3: Posterior means (black dots) and quantiles of order 0.025 and 0.975 (grey triangles) of parameters of model $\mathcal{M}_8$ and the log-likelihood when $I_0 \in \{1, 5, 10, 20, 30, \ldots, 100\}$. The value $I_0 = 10$ that is used in the main text is indicated by the vertical dashed red line. Parameters are the introduction date (t_0), the initial population size (N_0), the mortality rate (ρ), the infection strength (w), the proportion of the observable compartment (ϕ), the proportions of symptomatic hosts in the observable compartment (γ_1 and γ_2), the parameters governing the preference in sampling at-risk hosts (β_1 and β_2), and the false-negative rate (ϵ).

Model	Posterior statistic	t_0	N_0 ($\times 10^6$)	ρ	w_{12}	ϕ	γ_1	γ_2	β_1	β_2 ($\times 10^{-3}$)	ϵ
$\mathcal{M}_3$	Mean	-170	3.5	0.016	0.056	1	0.79	0.52	0.0070	0.087	0.042
	Median	-170	3.1	0.016	0.055	1	0.80	0.52	0.0058	0.056	0.031
	0.025-quantile	-210	1.1	0.008	0.050	1	0.57	0.26	0.0019	0.002	0.001
	0.975-quantile	-130	8.3	0.022	0.072	1	0.94	0.79	0.0193	0.358	0.140
$\mathcal{M}_8$	Mean	-360	4.7	0.016	0.055	0.012	0.81	0.56	0.59	8.0	0.042
	Median	-370	4.1	0.016	0.054	0.008	0.83	0.56	0.61	4.5	0.031
	0.025-quantile	-270	1.6	0.009	0.050	0.002	0.60	0.29	0.10	0.1	0.001
	0.975-quantile	-440	10.9	0.021	0.067	0.049	0.95	0.81	0.99	35.0	0.141

Table S4: Posterior means, medians, 0.025–quantiles and 0.975–quantiles of parameters of models $\mathcal{M}_3$ and $\mathcal{M}_8$.

Model	Posterior statistic	Introduction year	Number of infected hosts			Proportion of infected hosts		
			Whole pop.	Observable	Hidden	Whole pop.	Observable	Hidden
$\mathcal{M}_3$	Mean	2001	840	840	0	0.00030	0.00030	0
	Median	2001	760	760	0	0.00025	0.00025	0
	0.025-quantile	1998	430	430	0	0.00008	0.00008	0
	0.975-quantile	2005	1650	1650	0	0.00084	0.00084	0
$\mathcal{M}_8$	Mean	1985	0.23×10^6	880	0.23×10^6	0.049	0.028	0.049
	Median	1987	0.19×10^6	830	0.19×10^6	0.049	0.028	0.050
	0.025-quantile	1978	0.03×10^6	490	0.03×10^6	0.009	0.005	0.009
	0.975-quantile	1993	0.66×10^6	1630	0.66×10^6	0.092	0.053	0.092

Table S5: Posterior means, medians, 0.025–quantiles and 0.975–quantiles of the introduction year, and the number / proportion of infected hosts in December 2016 (in the whole host population, in the observable compartment and in the hidden compartment) under models $\mathcal{M}_3$ and $\mathcal{M}_8$.

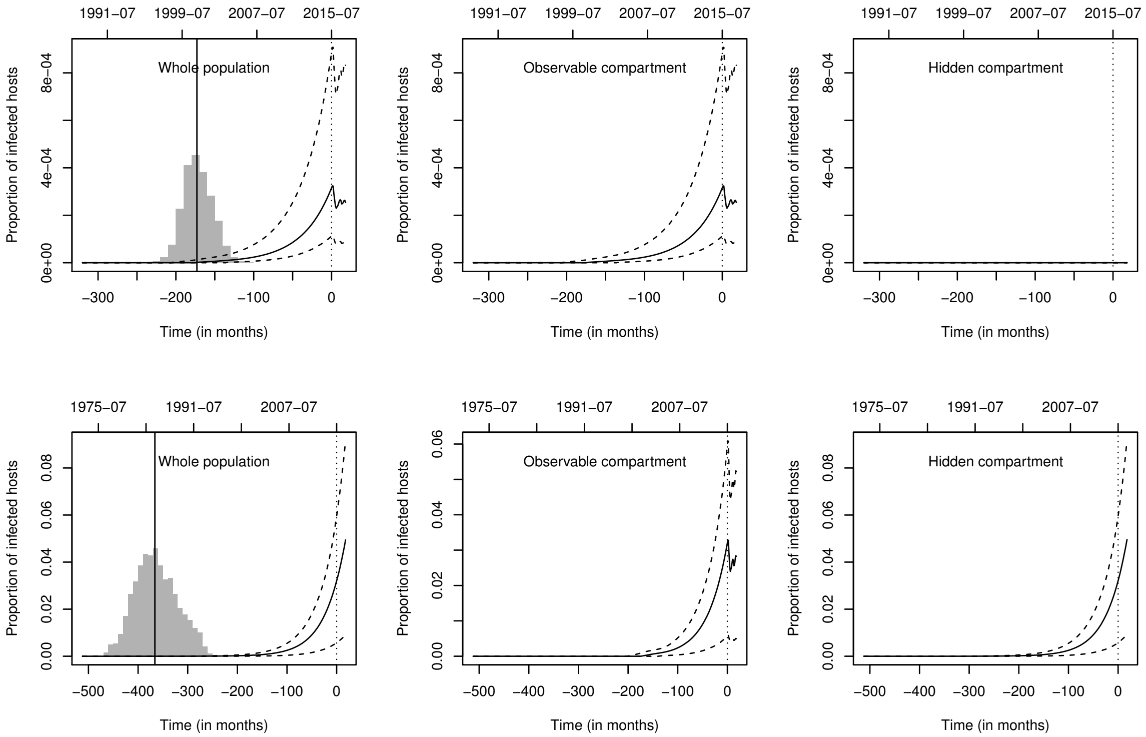

Figure S4: Posterior medians, 0.025-quantiles and 0.975-quantiles of the past proportions of infected hosts in the whole host population (left), the observable compartment (center) and the hidden compartment (right) under models $\mathcal{M}_3$ (top) and $\mathcal{M}_8$ (bottom); the median is given by the continuous curve, the quantiles by the dashed curves. The prevalence in the hidden compartment is zero under model $\mathcal{M}_3$ since this compartment is empty. On the left panels, the grey histograms and the continuous vertical line give the posterior distributions of the introduction date and its posterior median under each model. The dotted vertical line gives the date of the first observation.

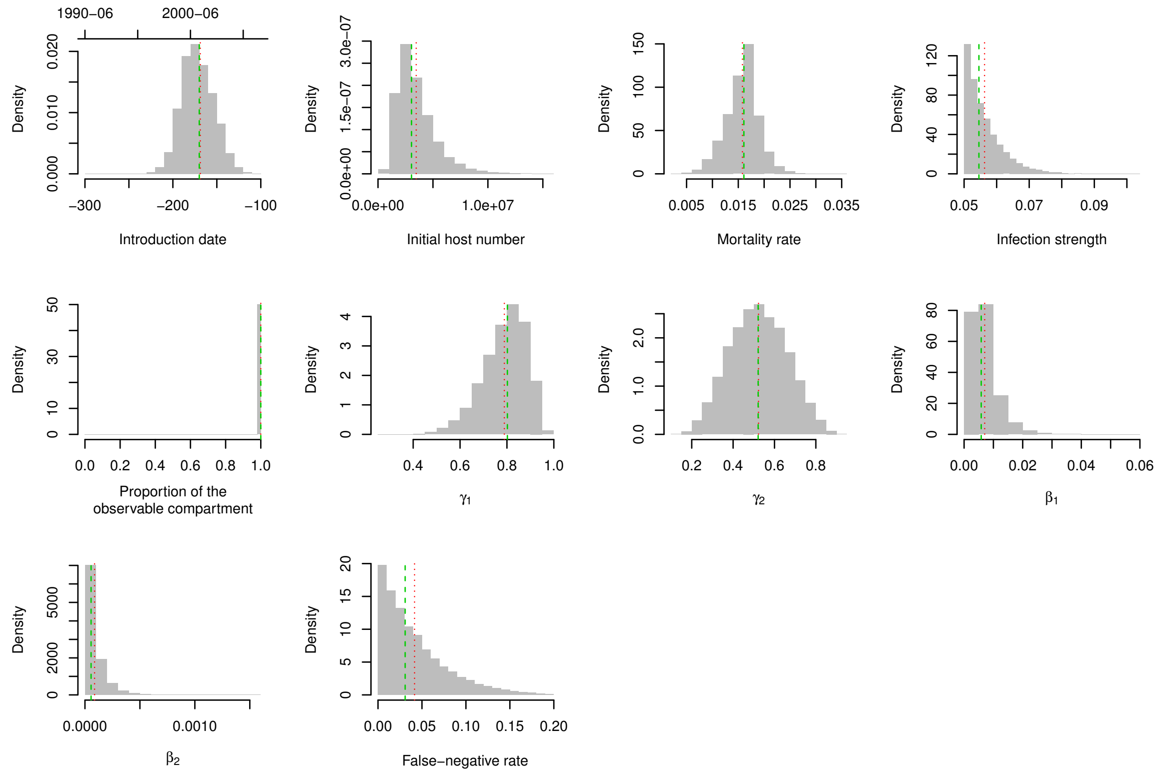

Figure S5: Marginal posterior distributions of the parameters of model $\mathcal{M}_3$: introduction date (t_0), initial population size (N_0), mortality rate (ρ), infection strength (w), proportion of the observable compartment (ϕ), proportions of symptomatic hosts in the observable compartment (γ_1 and γ_2), parameters governing the preference in sampling at-risk hosts (β_1 and β_2), false-negative rate (ϵ). Vertical dotted line: posterior mean; vertical dashed line: posterior median.

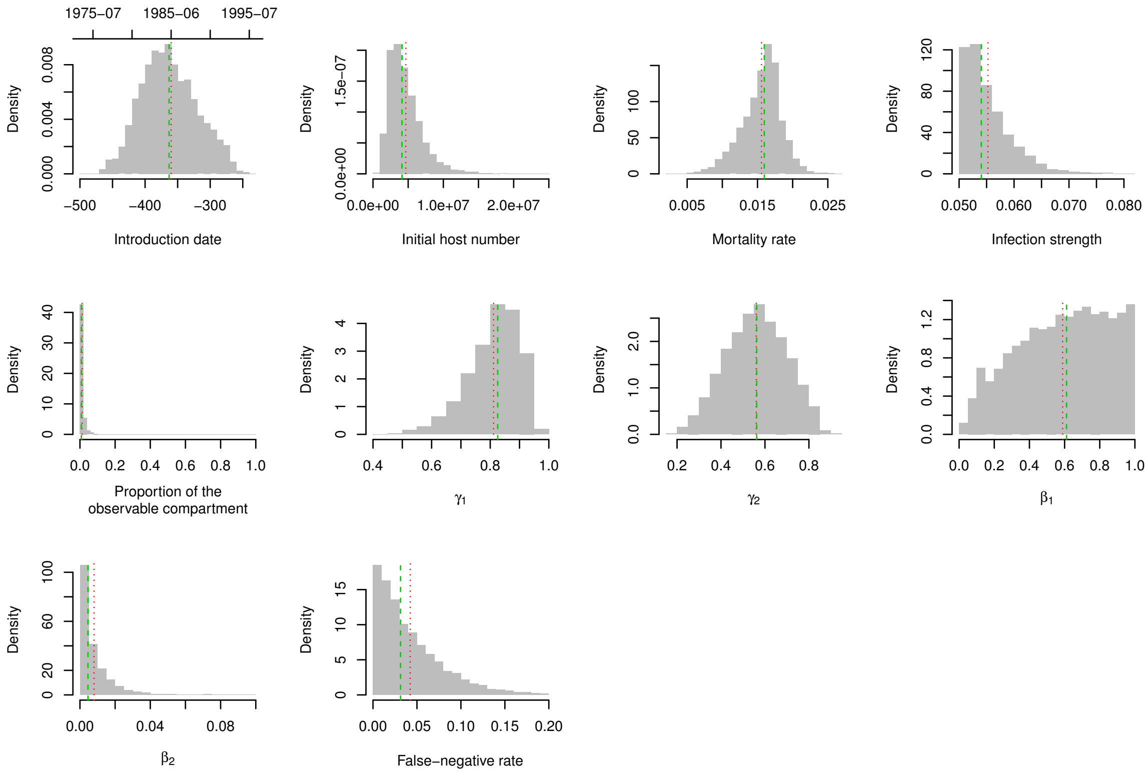

Figure S6: Marginal posterior distributions of the parameters of model $\mathcal{M}_8$: introduction date (t_0), initial population size (N_0), mortality rate (ρ), infection strength (w), proportion of the observable compartment (ϕ), proportions of symptomatic hosts in the observable compartment (γ_1 and γ_2), parameters governing the preference in sampling at-risk hosts (β_1 and β_2), false-negative rate (ϵ). Vertical dotted line: posterior mean; vertical dashed line: posterior median.

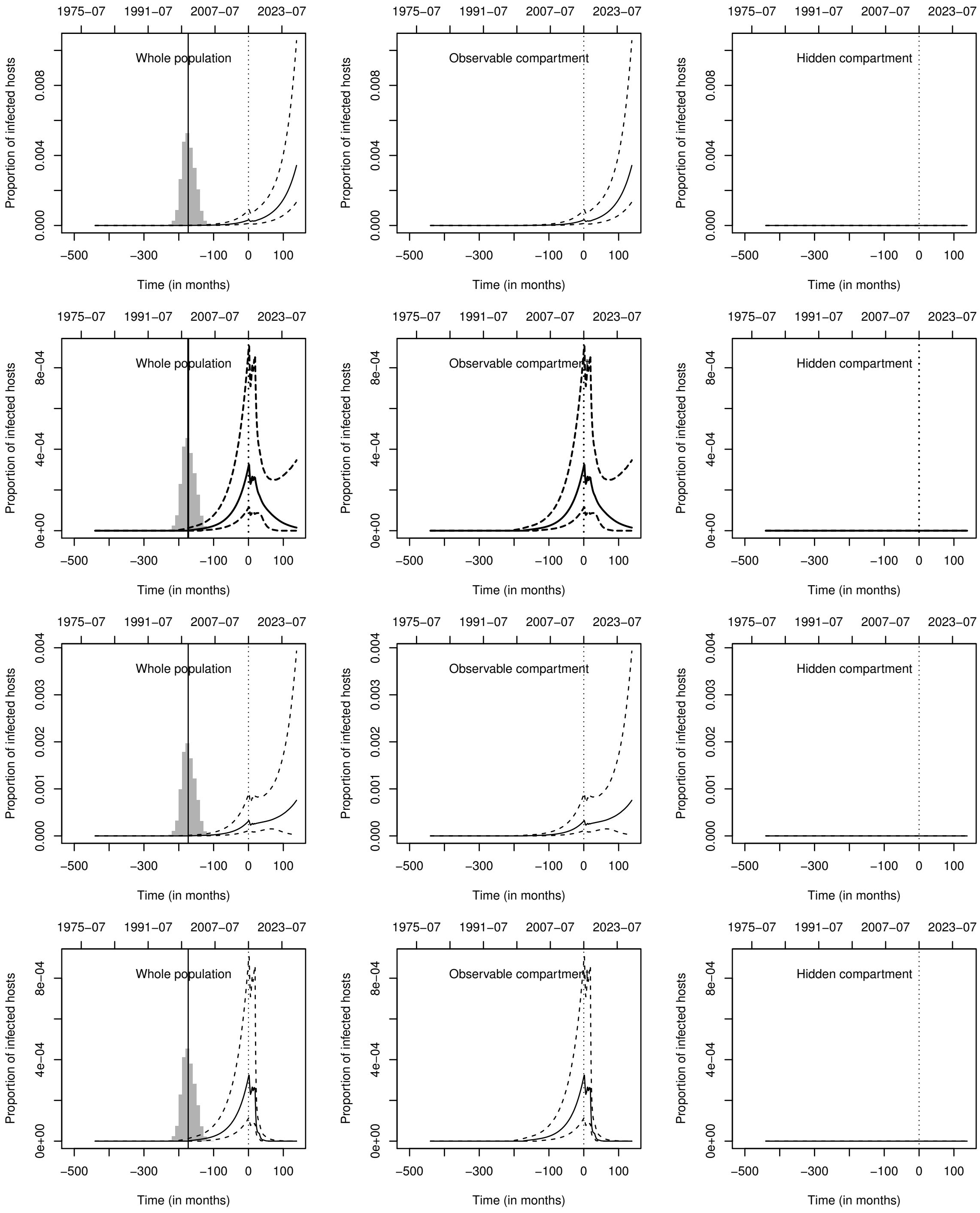

Figure S7: Posterior medians, 0.025-quantiles and 0.975-quantiles of the past and future proportions of infected hosts in the whole host population (left), the observable compartment (center) and the hidden compartment (right) under model $\mathcal{M}_3$. Each line correspond to different surveillance characterizations in the prediction part of the curves: the numbers of symptomatic and asymptomatic plants and Pref(t) are equal to (80,20,0.995) in the 1st line, (800,200,0.995) in the 2nd line, (80,20,0.999) in the 3rd line, (800,200,0.999) in the 4th. See Supplementary Figure SS4 for additional details on plot construction.

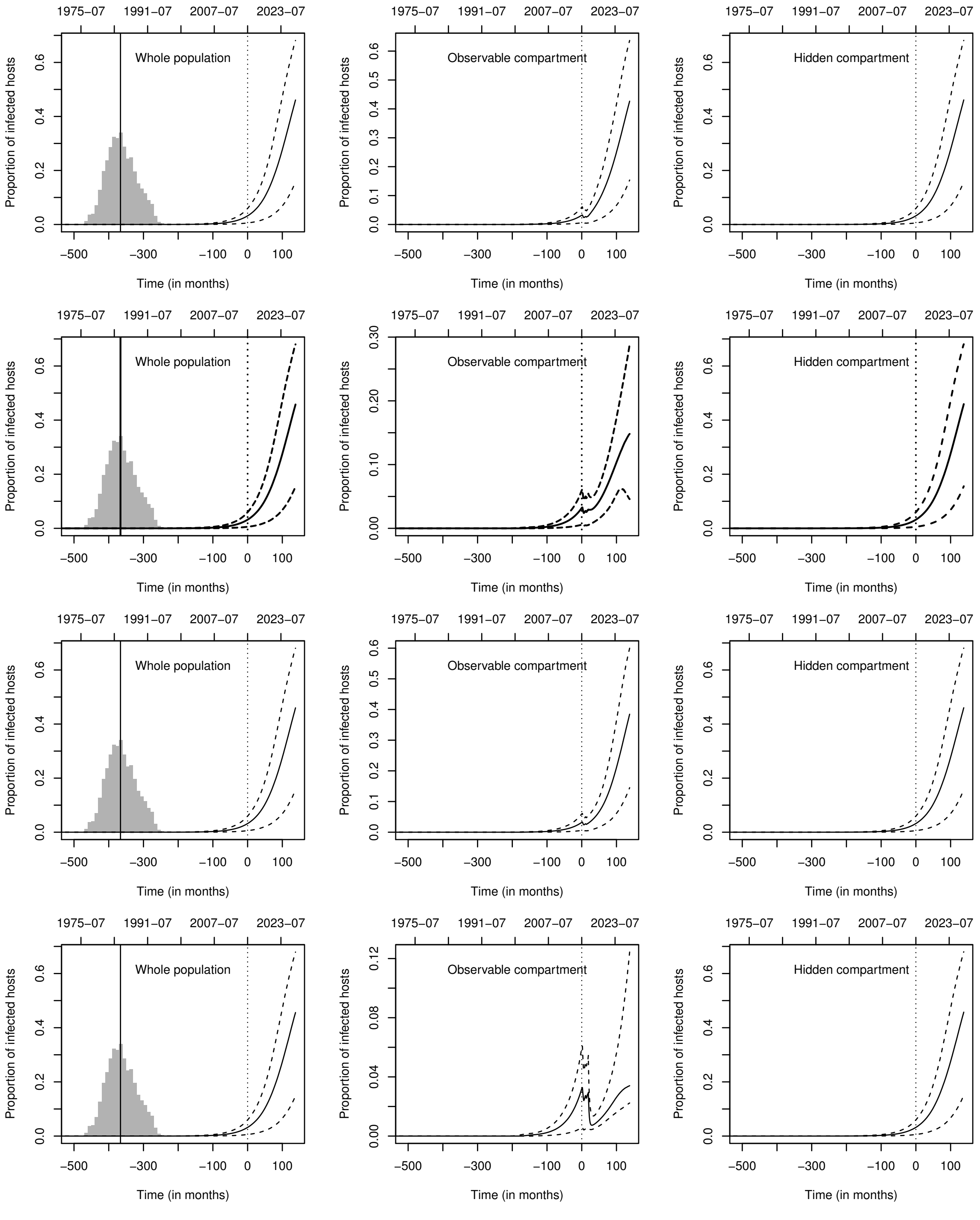

Figure S8: Posterior medians, 0.025-quantiles and 0.975-quantiles of the past and future proportions of infected hosts in the whole host population (left), the observable compartment (center) and the hidden compartment (right) under model $\mathcal{M}_8$. Each line correspond to different surveillance characterizations in the prediction part of the curves: the numbers of symptomatic and asymptomatic plants and Pref(t) are equal to (80,20,0.6) in the 1st line, (800,200,0.6) in the 2nd line, (80,20,0.9) in the 3rd line, (800,200,0.9) in the 4th. See Supplementary Figure SS4 for additional details on plot construction.
